# Suboptimal codon pairs trigger ribosome collisions and cellular quality control responses in tRNA modification mutants

**DOI:** 10.1093/nar/gkaf1311

**Published:** 2025-12-23

**Authors:** Jie Wu, Cristian Eggers, Olga Sin, Łukasz Koziej, Hector Mancilla, Fabienne Mollet, Hans R Schöler, Hannes C A Drexler, Tristan Ranff, Christian Fufezan, Claudine Kraft, Sebastian Glatt, Jan M Bruder, Sebastian A Leidel

**Affiliations:** Department of Chemistry, Biochemistry and Pharmaceutical Sciences (DCBP), University of Bern, Bern 3012, Switzerland; The Graduate School for Cellular and Biomedical Sciences (GCB), University of Bern, Bern 3012, Switzerland; Department of Chemistry, Biochemistry and Pharmaceutical Sciences (DCBP), University of Bern, Bern 3012, Switzerland; The Graduate School for Cellular and Biomedical Sciences (GCB), University of Bern, Bern 3012, Switzerland; Department of Chemistry, Biochemistry and Pharmaceutical Sciences (DCBP), University of Bern, Bern 3012, Switzerland; Malopolska Centre of Biotechnology (MCB), Jagiellonian University, Kraków 30387, Poland; Department for Biological Sciences and Pathobiology, University of Veterinary Medicine Vienna, Vienna 1210, Austria; Institute of Biochemistry and Molecular Biology (ZBMZ), Faculty of Medicine, University of Freiburg, Freiburg 79104, Germany; Department of Chemistry, Biochemistry and Pharmaceutical Sciences (DCBP), University of Bern, Bern 3012, Switzerland; Department for Cell and Developmental Biology, Max Planck Institute for Molecular Biomedicine, Muenster 48149, Germany; Bioanalytical Mass Spectrometry Unit, Max Planck Institute for Molecluar Biomedicine, Muenster 48149, Germany; Faculty of Engineering Sciences, Heidelberg University, Heidelberg 69120, Germany; Faculty of Engineering Sciences, Heidelberg University, Heidelberg 69120, Germany; Institute of Biochemistry and Molecular Biology (ZBMZ), Faculty of Medicine, University of Freiburg, Freiburg 79104, Germany; Centre for Integrative Biological Signalling Studies (CIBSS), University of Freiburg, Freiburg 79104, Germany; Malopolska Centre of Biotechnology (MCB), Jagiellonian University, Kraków 30387, Poland; Department for Biological Sciences and Pathobiology, University of Veterinary Medicine Vienna, Vienna 1210, Austria; Department for Cell and Developmental Biology, Max Planck Institute for Molecular Biomedicine, Muenster 48149, Germany; Department of Chemistry, Biochemistry and Pharmaceutical Sciences (DCBP), University of Bern, Bern 3012, Switzerland

## Abstract

Transfer RNA (tRNA) modifications tune translation rates and codon optimality, thereby optimizing co-translational protein folding. However, the mechanisms by which tRNA modifications modulate codon optimality and trigger phenotypes remain unclear. Here, we show that ribosomes stall at specific modification-dependent codon pairs in wobble uridine modification (U_34_) mutants. This triggers ribosome collisions and a coordinated hierarchical response of cellular quality control pathways. High-resolution ribosome profiling reveals an unexpected functional diversity of U_34_ modifications during decoding. For instance, 5-carbamoylmethyluridine (ncm^5^U) exhibits distinct effects at the A and P sites. Importantly, ribosomes only slow down at a fraction of codons decoded by hypomodified tRNA, and the decoding speed of most codons remains unaffected. However, the translation speed of a codon largely depends on the identity of A- and P-site codons. Stalling at modification-dependent codon pairs induces ribosome collisions, triggering ribosome-associated quality control (RQC) and preventing protein aggregation by degrading aberrant nascent peptides and messenger RNAs. Inactivation of RQC stimulates the expression of molecular chaperones that remove protein aggregates. Our results demonstrate that loss of tRNA modifications primarily disrupts translation rates of suboptimal codon pairs, showing the coordinated regulation and adaptability of cellular surveillance systems. These systems ensure efficient and accurate protein synthesis and maintain protein homeostasis.

## Graphical Abstract

**Graphical Abstract:**
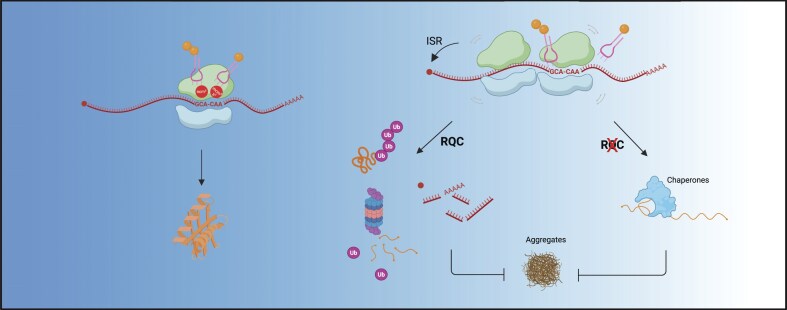

## Introduction

Translation speed varies along individual messenger RNA (mRNA) transcripts, thereby substantially impacting protein synthesis rates and orchestrating co-translational protein folding and protein localization [1–7]. To explain such differences in translation dynamics, different approaches have been used. The concept of codon optimality applies a supply-and-demand model for transfer RNAs (tRNAs) and their respective codons and best explains codon-specific translation rates [8]. However, a major limitation is that an identical optimality value is assigned to all instances of a codon across the transcriptome, and it is unclear how the numerous factors known to affect translation elongation rates, such as tRNA modifications, mRNA secondary structure, adjacent codons, or the position of amino acids in nascent peptides modulate individual codons [4, 5, 9–12]. Thus, the extent to which context-specific factors tune the optimality of individual codons and how this alters local translation dynamics, remains an open question.

tRNA modifications are crucial for tuning codon optimality and codon-specific translation dynamics because tRNA selection largely depends on codon-anticodon interactions in the A site of translating ribosomes. While most of these interactions adhere to Watson–Crick rules, wobble pairing allows cells to decode multiple codons with a single tRNA. The physicochemical parameters of such base-pairing interactions depend on RNA modifications in the anticodon loop [13, 14]. A key example are wobble uridines at tRNA position 34 (U_34_). In eukaryotes, U_34_ nucleotides are generally modified by 5-carbamoylmethyluridine (ncm^5^U), 5-methoxycarbonylmethyluridine (mcm^5^U), or 5-methoxycarbonylmethyl-2-thiouridine (mcm^5^s^2^U) by the Elongator (Elp) complex and the Urm1 pathway [15–18]. These highly conserved modifications are critical for decoding [19, 20] and their absence is associated with several human diseases, including cancers, familial dysautonomia, and DREAM-PL syndrome [21–26]. Mechanistically, loss of U_34_ modifications slows the decoding of specific codons and thereby alters codon optimality in different species [9, 10, 26–29]. Interestingly, U_34_-modification mutants exhibit widespread aggregation of cellular proteins, similar to strains lacking co-translational chaperones [10, 30, 31]. This phenotype emphasizes the link between translation dynamics and protein quality. Furthermore, perturbations in translation dynamics due to defective components of the translation machinery or mRNA, or environmental stress can stall ribosomes and induce ribosome collisions. Collided ribosomes trigger different cellular quality control pathways like ribosome-associated quality control (RQC) that leads to the degradation of potentially defective mRNA by no-go decay (NGD) and the integrated stress response (ISR) to reduce initiation rates avoiding further collisions [32–37]. To activate RQC, the E3-ubiquitin ligase Hel2 recognizes the extended interface formed by collided ribosomes [35, 38–41]. Previous studies have shown that strong perturbations of translation, such as ultraviolet, drug-induced stress, or artificial reporter constructs, induce ribosome collisions and trigger different branches of cellular quality control [37]. In contrast, codon-specific slowdowns in tRNA modification mutants are comparably subtle and much closer to physiological conditions. Hence, it is not clear whether they are sufficient to trigger ribosomal collisions and how these defects trigger cellular quality control.

**Figure 1.**
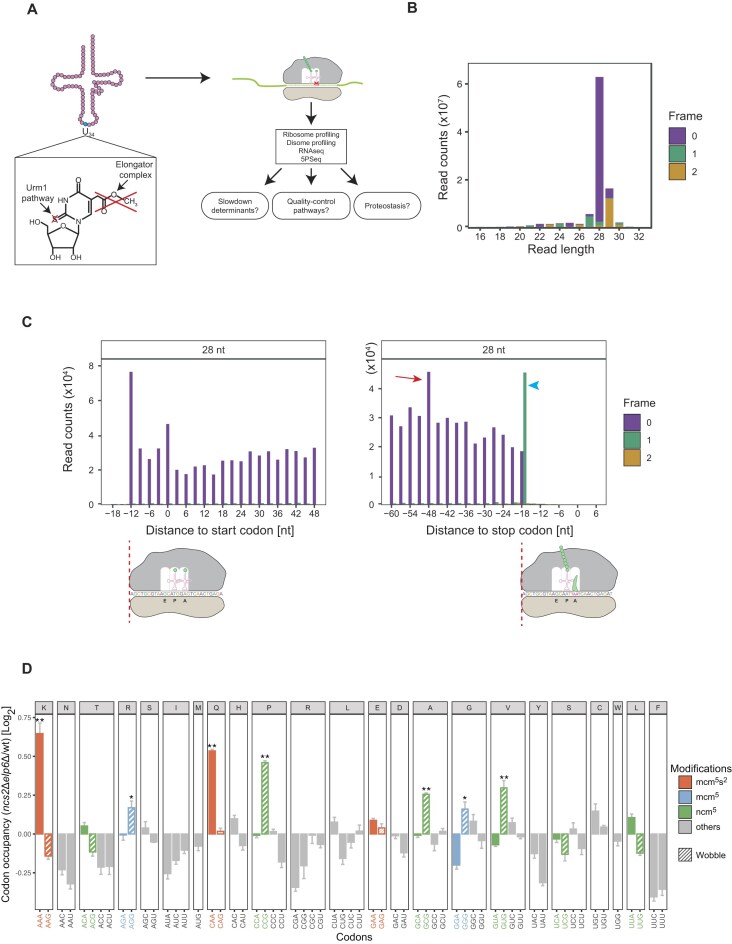
_34_ modifications affect translation differentially. (**A**) Overview of this study. mcm^5^s^2^U (chemical formula shown), mcm^5^U, and ncm^5^U are located at wobble uridine (U_34_). Deleting *NCS2* (Urm1 pathway) and *ELP6* (Elongator complex) completely removes these modifications. Ribosome profiling, disome profiling, RNAseq, and 5PSeq were performed. (**B**) The majority of monosome footprints were 28 nt long and predominantly in reading frame 0. (**C**) The distribution of the 5′ end of 28-nt footprints is shown at the start (left) and stop (right) codon; the cartoon below depicts initiating and terminating ribosomes. The terminating ribosomes accommodate four nucleotides in the ribosomal A site due to eRF1 recognition, which is visible as an apparent frameshift at the stop codon (blue arrowhead). A peak 48 nt upstream of the stop codon indicates ribosomes that collide with terminating ribosomes (red arrow). (**D**) Alterations of codon occupancy in the absence of U_34_ tRNA modifications. Relative A-site occupancy of each codon in *ncs2*∆*elp6*∆ mutant cells compared to wild type. Three biological replicates we used for both genotypes. Log_2_ fold change >0 indicates that codons are decoded slow in the mutant. Modification-dependent codons are highlighted by color: mcm^5^s^2^U (red); mcm^5^U (blue); ncm^5^U (green). Significantly slow codons with log_2_ fold change > 0.1 are highlighted with asterisks (one-sided Welch’s *t*-test; *P-value <0.05, **P-value <0.01).

To determine how sequence context tunes the optimality of individual codons across the transcriptome and how this modulates cellular quality control, we applied high-resolution ribosome profiling and disome sequencing in baker’s yeast devoid of U_34_ modifications (Fig. 1A) [42–46]. We found that the previously underappreciated ncm^5^U and mcm^5^U modifications are required for wobble decoding. Surprisingly, ncm^5^U plays a strikingly different role at the P site; loss of this modification exacerbates slow A-site decoding when the P site contains a codon cognate to ncm^5^-modified tRNAs. Importantly, while most suboptimal codons are translated without detectable effect, specific U_34_-dependent codon pairs induce ribosomal collisions and trigger RQC. Activation of RQC by Hel2 enhances the degradation of mRNAs enriched in modification-dependent codon pairs, thereby contributing to the maintenance of cellular protein homeostasis. RQC targets are enriched in suboptimal codon pairs but less likely to encode aggregation-prone proteins. When RQC is inactive, cells upregulate molecular chaperones via Hsf1 and are better able to cope with proteotoxic stress. Our findings demonstrate that codon pairs are critical to define codon optimality and that RQC coordinates with the cellular chaperone machinery in a hierarchical manner to maintain cellular protein homeostasis in response to codon-specific translation defects.

## Materials and methods

### Yeast strains and growth conditions

Experiments were performed in S288c (BY4741) yeast. All yeast strains and plasmids are listed in Supplementary Tables S4 and S5. Yeast cultures were grown to mid-exponential phase OD_600_ 0.4–0.5 at 30°C, 200 rpm in YPD (Formedium).

### Ribosome profiling

Ribosome profiling was performed as described with minor modifications [45, 46]. Yeast cells were harvested by vacuum filtration and the pellet snap frozen in liquid nitrogen with droplets of lysis buffer (ribosome profiling: *n* = 3 for wild type and *ncs2*∆*elp6*∆; *n* = 2 for *hel2*∆ and *ncs2*∆*elp6*∆*hel2*∆; disome profiling: *n* = 2 for wild type, *hel2*∆, *ncs2*∆*elp6*∆, and *ncs2*∆*elp6*∆*hel2*∆). The mixture of cells and buffer was lysed using a freezer mill (Spex SamplePrep 6770). The resulting powder was thawed in a water bath and cleared by two rounds of centrifugation (3000 × *g* for 3 min at 4°C and 10 000 × *g* for 5 min at 4°C). A_260_ absorbance of the lysate was measured, and 8 U of RNA were digested with 800 U RNaseI (AM2294, Invitrogen) in 80 µl reaction volume at 22°C and 1400 rpm for 1 h.

Digested samples were loaded on a 10%–50% sucrose gradient and separated by ultracentrifugation at 35 000 rpm, 4°C for 3 h using a SW41 rotor (Beckman Coulter). Sucrose gradients were fractionated using a Piston Gradient Fractionator (Biocomp). Both monosome and disome fractions were collected. Sodium dodecyl sulphate (SDS) was added to a final concentration of 1% prior to snap freezing the fractions. RNA was extracted from the fractions using hot acid phenol–chloroform. Ten micrograms of RNA per sample was size-separated on a 15% Tris-Borate-EDTA (TBE)–urea polyacrylamide gel. For monosomes, the gel between the 28 and 32 nt marker was excised and for disomes between 56 and 64 nt. The excised bands were crushed in a 1.5 ml microtube with 400 µl elution buffer [0.3 M NaOAc, pH 5.5, 1 mM ethylenediaminetetraacetic acid (EDTA), pH 8.0, 0.1 U/µl SuperaseIN (AM2694, Invitrogen)] and eluted overnight at 4°C on a spinning wheel. The next day, the gel pieces were removed using a Spin-X column (CLS8162, Merck, Corning Costar Spin-X centrifuge tube filters) and RNA was precipitated overnight at −80°C.

Ribosome-protected fragments were dephosphorylated using T4 Polynucleotide Kinase (M0201, New England Biolabs). Dephosphorylated RNA was extracted using ROTI Acqua P/C/I for RNA extraction (X985.1, Carl Roth) and RNA precipitated with NaOAc overnight at −80°C. Subsequently, RNA was ligated using T4 RNA ligase 2 (M0242, New England Biolabs) to a preadenylated linker and purified with ROTI Aqua P/C/I for RNA extraction and precipitated with NaOAc. Samples were reverse transcribed using SuperScript III (18080093, Invitrogen). Subsequently, template RNA was hydrolyzed using NaOH. Complementary DNA (cDNA) was run on a 10% TBE–urea polyacrylamide gel. The band corresponding to the cDNA was excised and crushed in 360 µl H_2_O and eluted for 20 min in a thermoblock at 70°C and 1400 rpm. Gel pieces were removed using a Spin-X column and the cDNA precipitated with NaOAc overnight at −80°C and resuspended in 11 µl H_2_O. cDNA was circularized with CircLigase II (CL9021, Lucigen). Libraries were polymerase chain reaction (PCR) amplified using Phusion DNA Polymerase (M0530, New England Biolabs). Quality assessment of the libraries and sequencing were performed by the NGS platform of the University of Bern.

### mRNA and 5P sequencing

Total RNA extraction was performed as described in [10]. A fraction of the cells harvested for Ribosome profiling was used for mRNAseq. The frozen pellets were resuspended in lysis buffer (50 mM NaOAc, pH 5.5, 10 mM EDTA, 1% SDS) and cells were lysed using a Fastprep24 bead beater. Lysates were clarified by centrifugation. Samples were treated with 100 µg/ml Proteinase K (AM2546, Invitrogen) for 20 min at 60°C. Finally, RNA was extracted with acid-phenol/chloroform. Library preparation was performed using a TruSeq-Stranded-mRNA kit (2002594, Illumina) according to the manufacturer’s instructions. 5Pseq libraries were generated as described in [47].

### Fluorescence measurement of the reporter

A total of 2–3 OD_600_ of yeast cultures of OD_600_ 0.6–0.8 were pelleted and resuspended in lysis buffer [20 mM MES, 100 mM NaCl, 30 mM Tris–HCl, pH 7, 5, 1% Triton X-100, supplemented with 1× cOmplete protease inhibitor (4693159001, Merck)]. Cells were lysed using a Fastprep24 bead beater and the lysate was cleared by centrifugation. Green fluorescent protein (GFP) and mKate fluorescence was measured using an Infinite M1000 plate reader (Tecan).

### 35S labeling

A total of 3 OD_600_ units of yeast during exponential growth were inoculated to 3 ml SC–Met medium containing glucose and 15 μCi/ml ^35^S L-methionine. Five hundred microliter aliquots were taken at timepoints 0, 10, and 30 min. Cells were pelleted and resuspended in 500 ml ice-cold 20% trichloroacetic acid (TCA), incubated on ice for 20 min, precipitated, and washed with acetone. Samples were run on a 4%–12% gradient gel (mPAGE, Sigma–Aldrich) and radioactive signals were scanned using a TyphoonFLA1000 Phosphoimager. Bands were quantified using AzureSpot Analysis Software (Azure Biosystems).

### Live-cell imaging

Yeast cells were grown in log-phase in high-cell-density (HCD) medium (monosodium glutamate 4.5 mg/ml, yeast nitrogen base 12 mg/ml, inositol 1.8 µg/ml, 20 mM 2-(*N*-morpholino)ethanesulfonic acid (MES) buffer, pH 6.0, 6% glucose, and amino acids) [48] at 30°C. Overnight cultures were diluted to an OD_600_ of 0.3 and further incubated until OD_600_ = 0.6. To monitor the translocation of Hsf1-GFP to the nucleus, cultures were treated with 2 mM diamide for 1 h. For nuclear staining, the samples were incubated with 100 nM Hoechst reagent (H-1399, Invitrogen) for 5 min. Cells were then pelleted at 3000 × *g* for 3 min and resuspended in fresh HCD medium. Yeast cells were attached to 35 mm glass bottom dishes (D35-20, 1.5, In Vitro Scientific) pretreated with concanavalin A type IV (1 mg/ml, Sigma–Aldrich) and imaged at room temperature. Fluorescent microscopy images were recorded with a DeltaVision Ultra High-Resolution Microscope with a UPlanSApo 100 ×/1.4 oil Olympus objective, using a sCMOS pro.edge camera (GE Healthcare, Applied Precision). Image analysis was performed using FIJI [49]. Images from each figure panel were taken with the same imaging setup and are shown with identical contrast settings. All images were generated by collecting a z-stack of 25 pictures with focal planes 0.20 μm apart. Single focal planes of representative images are shown.

### High-content microscopic Hsp104 assay

Yeast cells expressing *HSP104-GFP* from the endogenous locus were grown in synthetic-defined complete medium until reaching OD_600_ ∼0.6. The cells were then diluted to an OD_600_ = 0.1 and 20 µl of the diluted culture were seeded to each well of a 384-well plate with a transparent bottom.

Cell suspensions were centrifuged at 2000 × *g* for 1 min to form a monolayer. Subsequently the plate was imaged on an Operetta High Content Analysis System (PerkinElmer) for 4 h. Images were captured every 10 min for the first 90 min and subsequently every 30 min. Two different fields of view (three planes each) were captured with digital phase contrast and GFP channels for each well and time point using a 60× high NA air objective. The images were quantified using Harmony Software v4.1, Revision 128972 (PerkinElmer). Briefly, all planes were stacked, and cells were filtered based on their roundness and texture using the phase-contrast images, as well as GFP background intensity. For quantification of GFP-positive spots, size, intensity, and relative contrast to the surrounding area were taken into account. For each condition, between 500 and 1000 cells were imaged at time point T0.

### Cryo-electron microscopy (Cryo-EM) grid preparation

Monomeric 80S ribosomes were isolated by using sucrose gradient purification after MNase treatment. Following MNase digestion and sucrose gradient centrifugation, 80S monosome peaks from wild-type or *ncs2*∆*elp6*∆ yeast were concentrated at 4°C with a Amicon Ultra 100 kDa MWCO 0.5 ml centrifugal filter, to the final concentration of approximately A_260_∼20 and A_280_∼10 in 50 mM Tris–HCl, pH 7.5, 50 mM NH_4_Cl, 50 mM KCl, 7 mM MgCl_2_, and 1 mM dithiothreitol (DTT). Approximately 3.5 μl of the ribosome concentrate was applied onto freshly glow-discharged and graphene oxide-coated TEM grids (Quantifoil R2/1, Cu 200 mesh) and plunge-frozen into liquid ethane by a Vitrobot Mark IV (Thermo Fisher Scientific) using the following parameters: humidity 100%, temperature 4°C, blot total 1, wait time 0, blot force 0, blot time 2 s, drain time 0 s. For each strain two samples/grids (WT#1, WT#2, *ncs2*∆*elp6*∆#1, *ncs2*∆*elp6*∆#2) were prepared from independent ribosome isolations (Supplementary Table S1).

### Cryo-EM single-particle reconstruction

Cryo-EM datasets were collected at the National Cryo-EM Centre SOLARIS (Kraków, Poland). The datasets contained 8595 (WT#1), 10 009 (WT#2), 9486 (*ncs2*∆*elp6*∆#1), and 8583 (*ncs2*∆*elp6*∆#2) movies. The movies were acquired using a Titan Krios G3i microscope (Thermo Fisher Scientific) operated at 300 kV accelerating voltage, magnification of 105k, and corresponding pixel size of 0.8456 Å/px. A K3 direct electron detector used for data collection was fitted with BioQuantum Imaging Filter (Gatan) using a 20 eV slit. The K3 detector was operated in counting mode. Imaged areas were exposed to 40 e^−^/Å^2^ total dose (corresponding to ∼16 e^−^/px/s dose rate measured in vacuum). Forty-frame movie stacks were obtained using under-focus optical conditions with a defocus range of −2.2 to −0.7 µm and 0.3 µm steps. The collected datasets were analyzed using cryoSPARC v4.4.1 [50]. First, patch motion correction and patch CTF estimation steps were performed. Next, a template picking based on a consensus *Saccharomyces cerevisiae* 80S ribosome volume, and subsequent 2D classification, resulted in 858 868 (WT#1), 758 909 (WT#2), 976 463 (*ncs2*∆*elp6*∆#1), and 672 666 (*ncs2*∆*elp6*∆#2) particles, respectively. Following “Ab-initio Reconstruction” and “Homogenous Refinement”, the particles served as an input for 3D classification. To optimize class distribution for comparative analysis, the particles from four datasets (WT#1 + WT#2 + *ncs2*∆*elp6*∆*#*1 + *ncs2*∆*elp6*∆*#*2) were mixed into a common pool. First, “Heterogenous Refinement” was used on the common pool to remove particles corresponding to 60S complexes. Next, to focus the analysis on 80S translating ribosomes in different states, focused 3D classifications were performed using soft masks generated from regions occupied by E-, P-, and A-site tRNA, and EF2 (Supplementary Fig. S2C). After each class selection step, the particles originating from individual strains and replicates were enumerated using the intersection option of the Particle Sets tool. The final particle stacks were reextracted without binning for “Reference Based Motion Correction” in cryo-SPARC [51]. During final rounds of “Homogeneous Refinement”, the particles were subjected to “Defocus Refinement”, “Global CTF Refinement”, and “Edwald Sphere Correction” to generate high-resolution maps. Local map resolution was calculated using cryoSPARC (Supplementary Fig. S2D and E). The combined half-maps and corresponding refinement masks were used for DeepEMhancer sharpening [52]. The cryo-EM maps were displayed using ChimeraX version 1.7 [53].

### Protein aggregate purification

Protein aggregates were isolated as described in [30]. Fifty OD_600_ of exponentially growing cells were harvested by vacuum filter and immediately snap-frozen. The pellets were resuspended in 1 ml of lysis buffer (20 mM NaPi, pH = 6.8, 1 mM EDTA, 10 mM DTT, 0.1% Tween 20) supplemented with protease inhibitors (0.5 mM AEBSF, 10 µg/ml aprotinin, 0.5 mg/ml benzamidine, 20 µM leupeptin, 5 µM pepstatin A); 60 U of zymolyase T20 (Zymo Research) were added, and cells were incubated at 22°C for 20 min at 1400 rpm. Extracts were chilled on ice and sonicated eight times with 50% amplitude and duty cycle 50% with a FisherBrand 120 tip sonicator (Thermo Fisher). Cell debris was pelleted by centrifugation at 200 × *g*, 4°C for 20 min. Protein concentration of the supernatants was quantified using the Bio-Rad Protein Assay. To precipitate aggregates, 2 mg of total protein in 1 ml of lysis buffer were centrifuged at 16 000 × *g*, 4°C for 20 min. The supernatant was discarded and the pellet resuspended in washing buffer I (20 mM NaPi, pH = 6.8, 2% NP-40, protease inhibitor cocktail) and sonicated six times with amplitude 50%, duty cycle 50%, and precipitated again at 16 000 × *g*, 4°C for 20 min. The pellet was resuspended in washing buffer I, sonicated and precipitated as before. Finally, aggregates were resuspended in detergent-free washing buffer by sonicating four times with amplitude 35%, duty cycle 50%. Following precipitation, pellets were dissolved in Laemmli sample buffer containing 100 mM DTT and 8 M urea and boiled at 95°C for 5 min. Total protein extracts and aggregates were run on 4%–12% NuPAGE Bis–Tris gels (Life Technologies) in MES–SDS running buffer and gels were stained with AzureRed Fluorescent Protein Stain (Azure Biosystems). Gels were scanned using a Sapphire Biomolucar Imager (Azure Biosystems) and images quantified with AzureSpot (Azure Biosystems).

### Proteomic analysis of collided ribosomes

50 OD_600_ units of yeast during exponential growth were harvested by vacuum filtering and snap-frozen with droplets of lysis buffer [46]. The mixture of cells and buffer was lysed using a SamplePrep 6770 cryo-mill (Spex). The resulting powder was thawed in a water bath and cleared by two rounds of centrifugation. A_260_ absorbance of the lysate was measured, and 15 U of RNA were diluted 1:1 with 2 mM CaCl_2_ and digested with 100 U Micrococcal Nuclease (Thermo Scientific) at 22°C and 1400 rpm for 15 min. Digested samples were loaded on a 10%–50% sucrose gradient and separated by ultracentrifugation at 35 000 rpm, 4°C for 3 h using a SW41 rotor (Beckman Coulter). Sucrose gradients were fractionated using a Piston Gradient Fractionator (Biocomp). The disome peak was collected and proteins precipitated using TCA. Proteins captured on beads were subsequently processed for nano-liquid chromatography coupled to tandem mass spectrometric analysis on an Ultimate 3000 Orbitrap LUMOS (Thermo) using a Acquity CSH column (200 × 0.075 mm, 1.7 μm; Waters) with a 90-min acetonitrile gradient (2%–40%) at a flow rate of 220 nl/min. Further details on the proteomic analysis can be found in the supplementary material.

### Sequencing data processing

Sequencing data from ribosome profiling was pre-processed by removing the adapter using the FastX_toolkit. Four randomized linker nucleotides at the 3′ end were trimmed. If the library was generated by dual randomization, three nucleotides at the 5′ end and six at the 3′ end were trimmed. The quality of data was evaluated using FastQC (v0.11.7). Processed reads were mapped to noncoding RNA, including ribosomal RNAs, tRNAs, small nucleolar RNAs (snoRNAs), and other annotated long non-coding RNAs (lncRNAs) using STAR (v2.5.3a) to remove nonprotein-coding reads [54]. Remaining reads were subsequently mapped to all annotated open reading frames in yeast using bowtie (v1.2.3) with unique mapping mode allowing at most one nucleotide mismatch with parameters “-v 1 -m 1 –norc –best –strata” [55]. Each open reading frame (ORF) was extended 21 bp at both 5′ end and 3′ end for the alignment of footprints around the start codons and stop codons. If an extra mismatched T was found at the 5′ end of the read, which is due to the extra insertion of an A by reverse transcriptase (RT) during the RT step, it was also removed. The processed data were used for downstream analysis.

The meta-analysis around the start codons or stop codons were performed by aligning the A of all AUG start codons at the 0 position or aligning the last nucleotide of all stop codons (UGA/UAA/UAG) at −1 position, respectively. Footprints in frames 0, 1, and 2 were defined, if the 5′ end of the read was mapped to the first, second, and third nucleotide of the codon, respectively. Footprints are grouped by read length to present the 3-nt periodicity. Due to the incomplete or excessive digest of RNaseI, the assignment of the A site for each read length is determined according to the first high peak at around −12 nt. In our study, we selected 28 nt footprints with frame 0 (A-site offset: 16 nt) and 29 nt footprints with frame 0 (A-site offset 16 nt) and frame 2 (A-site offset: 17 nt) for downstream analyses of codon translation rates.

Disome profiling data were pre-processed like monosome profiling. 56 to 64-nucleotide-long footprints are kept for analysis. Since no disome enrichment can be found around the start codon, when selecting the reads for downstream analysis, offsets were chosen according to the last disomes at the stop codons. RNA-seq reads were mapped to the coding sequences (CDS) without adaptor trimming.

### Calculation of the translation rate of codons

We used two independent methods to calculate translation speed. The location-based method bases its calculation on the read count for each codon on each transcript. It considers the information of the location of each codon in a specific transcript. To exclude potential biases are caused by codon location, we also used a read-based method, which depends on the total number of ribosome footprints across the transcriptome without retaining information on the location of a codon:

1. Read-based (Fig. 1D): The frequency of codons in the ribosomal A sites were quantified according to the length-specific offsets for all selected reads. The translation rate of sense 61 codons comparing mutant to wild type was calculated as [10] by normalizing the frequency of codons in the A sites to that in the +1, +2, +3 sites, followed by calculating the ratio of translation rate between mutant and wild type (15 codons were excluded at both end of CDS). To avoid that few extremely affected codons shift other codons away from the mean (log_2_ ratio = 0), the ratio of each codon is further normalized to the median of all ratios.
2. Position-based (Supplementary Fig. S1C) [56]: After assigning footprints to the A-site codon according to the selected offset, the read counts for each codon in the CDS can be quantified (15 codons at both 5′ and 3′ end of CDS were excluded). Only genes with read coverage of 0.1 reads per codon are included in the analysis. The pause score for each codon in each CDS is subsequently calculated by normalizing the read count of this codon to the average read count of all codons in this CDS:
  (1) \begin{eqnarray*}
\text{Pause}\ \text{score}\ = \frac{{Cij}}{{(\mathop \sum \nolimits_{i = l - 15}^{i = 15} Cij)/\left( {l - 30} \right)}},
\end{eqnarray*}

where Cij is the read count of codon i in gene j, l is the length of CDS (codons).

A vulnerability score of each codon in the mutant compared to wild type is calculated by normalizing the mutant pause score to that in wild type:


(2) \begin{eqnarray*}
\text{Vulnerability}\ \text{score}\ = \frac{{\text{pause}\ \text{score}\ \text{in}\ \text{mutant}}}{{\text{pause}\ \text{score}\ \text{in}\ \text{wild}\ \text{type}}}.
\end{eqnarray*}

To avoid dividing by zero during the calculation of the vulnerability score, we added 1 read to each position before calculating the pause score in the genes that were selected for analysis (0.1 reads per codon). The median of vulnerability scores of three replicates in mutant and wild type is used to represent the susceptibility of this codon to the absence of U_34_ modifications. A Student’s *t*-test is used to test the difference of pause scores between mutants and wild types. Significant slow codons are filtered with *P*-value <0.05.

### Motif analysis

Vulnerability scores of each of the 61 sense codons are ranked and the top 1000 of the codons with high vulnerability scores were selected for motif analyses. Motif analysis was performed for the 30 codons upstream of the codons of interest using kpLogo and probability logo is shown [57]. Positions with significant enriched or depleted residues are highlighted in red (Bonferroni-corrected *P*-value <0.01). To remove potentially embedded biases, the same analysis pipeline was implemented comparing two wild-type replicates. Upstream sequences of top 1000 codons were selected as the input of background sequence list in kpLogo.

### Di-codon analysis

The pause score for each codon pair in each ORF was calculated as in formula 1. The average of pause scores of each codon pair in the ORFs was used as translation rate of this codon pair. The ratio of averaged translation rates from three replicates between mutant and wild type were calculated as the alteration of the translation rate of codon pairs. To visualize the change of all codon pairs, codon pairs with ratio >2 are all assigned to 2 in the di-codon plot. In the di-codon plot, the size of circle represents the number of codon pairs in all CDSs in the analysis.

### Analysis of translation near codons of interest

All specific codon/codon pairs are aligned at position 0. The pause scores upstream or downstream of the codons/codon pairs of interest were averaged with all codon/codon pairs within that position [56]. Disome footprints are assigned to the position according to the location of the A site of the stalling ribosome (45 nt downstream of the 5′ end of the disome read).

### Analysis of disome enrichment

The monosome/disome footprints uniquely mapped to each ORF were quantified using a custom script. The differential expression analysis of disome enrichment was performed using DESeq2 (v1.28.1) [58]. Since the disome profile is positively correlated with the monosome profile, the expression quantified by monosome footprints are included in the differential expression (DE) analysis using design = ∼ sampleType + condition + condition:sampleType. The likelihood-ratio test was used to test a reduced model (∼ sampleType + condition) and the significant genes with disome enrichment or depletion were filtered using adjusted *P*-value <0.05. Genes with significantly enriched disomes in *ncs2*∆*elp6*∆ but significantly decreased disomes in *ncs2*∆*elp6*∆*hel2*∆ were selected as RQC targets. Genes with significantly depleted disomes in *ncs2*∆*elp6*∆ but significantly enriched disomes in *ncs2*∆*elp6*∆*hel2*∆ were selected as RQC-refractory transcripts (RRTs).

### DE analysis of RNA-seq data

The change of mRNA levels between strains was analyzed using DESeq2 (v1.28.1) [58]. Significant genes were tested using a Wald test and filtered with adjusted *P*-value <0.05. When performing differential-expression analysis between *ncs2*∆*elp6*∆*hel2*∆ and *ncs2*∆*elp6*∆ mutants, we sought to correct for changes in transcription that may be caused by the depletion of Hel2. Hence, we included RNA-seq of *hel2*∆ and wild-type strains in the analysis as in the analysis of disome enrichment. However, the results were not altered whether including *hel2*∆ in the analysis or not.

### Analysis of 5PSeq data

Adapter sequences were trimmed by cutadapt using parameters “-m 10 -a GATCGGAAGAGCACACGTCTGAACTCCAGTC” [59]. Untrimmed reads were also retained for downstream analysis due to the long insert size. The processed reads were mapped to noncoding RNA using STAR (v2.5.3a) [54]. The remaining reads were mapped to all annotated open reading frames in yeast using bowtie (v1.2.3) using the same parameters as for the analysis of ribosome profiling data [55]. PCR duplicates were further removed using UMI-tools with 8 nt randomized barcodes at the 5′ end of sequencing reads [60]. The 3-nt periodicity of reads was also evaluated. Unique mapped reads were quantified using a custom script. DE analysis was performed with DEseq2 (v1.28.1) by combining RNA-seq data using design = ∼ sampleType + condition + condition:sampleType [58]. The likelihood-ratio test was used to test a reduced model (∼ sampleType + condition). Log_2_ fold change of RQC-target and RRTs were compared between different strains.

### Additional computational analyses

The gene-ontology analysis was performed using the online Panther server on the gene-ontology resource (http://geneontology.org/). Genes for aggregate-related analysis are retrieved from [10]. Genes with fold change >2 between *ncs2*∆*elp6*∆ and wild type in aggregate proteomics are selected as aggregation-prone proteins and fold change <0.5 as non-aggregating proteins.

## Results

### U_34_ tRNA modifications differentially affect cognate and wobble decoding

Codon-specific translation defects of tRNA modification mutants have mostly been characterized globally and not at the level of individual transcripts, precluding an understanding of their context dependency and what underlies phenotypes of tRNA modification mutants. We therefore optimized our ribosome profiling protocol for improved A-site mapping and reading-frame homogeneity of the resultant footprints, allowing us to map translation dynamics at high resolution (Fig. 1B) [42, 43, 45, 46]. To verify the quality of our data, we aligned reads at the start and stop codon. First, we observed the expected 3-nt periodicity pattern reflecting ribosomal movements along mRNA (Fig. 1C and Supplementary Fig. S1A and B). Second, we observed a peak 48 nt upstream of the stop codon indicative of ribosomal queueing upstream of the terminating ribosome (48 nt = 28 + 2 + 18 nt) (Fig. 1C, right; red arrow). Finally, the footprints of terminating ribosomes (at ∼−18 nt relative to the stop codon) shift to a different reading frame within the same footprint length, consistent with the ribosomal A site accommodating 4 nt during eRF1 recognition (Fig. 1C and Supplementary Fig. S1A, right; blue arrowhead) [61–65]. These examples demonstrate that our optimized libraries capture subtle physiological translation events.

The absence of mcm^5^s^2^U in ${\mathrm{tRNA}}_{{\mathrm{UUG}}}^{{\mathrm{Gln}}}$ and ${\mathrm{tRNA}}_{{\mathrm{UUU}}}^{{\mathrm{Lys}}}$ slows down the decoding of their respective cognate CAA and AAA codons [9, 10], while no codon-specific translation defects have been linked to the loss of ncm^5^U and mcm^5^U. To obtain a more granular picture, we analyzed changes in codon-specific translation speed in *ncs2*Δ*elp6*Δ mutants using two independent normalization methods (position-based and footprint-based; see the ‘Materials and methods’ section). As expected, AAA and CAA are the most affected codons (Fig. 1D and Supplementary Fig. S1C) [9, 10]. However, we did not observe an effect on the translation speed of codons cognate to mcm^5^U-modified tRNAs (AGA and GGA) and ncm^5^U-modified tRNAs (ACA, CCA, GCA, GUA, UCA, and UUA; Fig. 1D and Supplementary Fig. S1C) showing that these modifications do not affect the pairing with the respective cognate tRNA. Unexpectedly, some of their synonymous G-ending codons (mcm^5^U: AGG and GGG; ncm^5^U: CCG, GCG, and GUG) slowed down significantly while the others (ncm^5^U: ACG, UCG, and UUG) did not change. To explain this difference, we assessed gene copy numbers of the different tRNA isoacceptors. The slow G-ending codons CCG and GCG rely on wobble decoding as they have no cognate tRNAs (Supplementary Fig. S1D) [66]. The remaining slow G-ending codons AGG, GGG, and GUG have cognate tRNAs. However, these tRNAs are not essential in yeast, demonstrating that these codons rely, at least in part, on wobble decoding by essential tRNAs [67]. In contrast, none of the codons that are synonymous to the mcm^5^s^2^U-modified tRNA (AAG, CAG, and GAG) are significantly slowed down. Hence codon-anticodon interactions of mcm^5^s^2^U differ from those of mcm^5^U and ncm^5^U. mcm^5^s^2^U facilitates decoding of cognate codons, while mcm^5^U and ncm^5^U optimize wobble decoding in the ribosomal A-site. Together, our results show that the previously underappreciated ncm^5^U and mcm^5^U modifications are required for U•G wobble pairing, but not for cognate decoding in the ribosomal A site. Thus, the decoding of U_34_ is more complex than previously thought.

A global slowdown in the decoding of specific codons might be caused by the arrest of ribosomes in a specific state during the elongation cycle [28, 68]. Therefore, we used single-particle cryo-EM to compare the overall distribution of ribosomal states in wild-type and *ncs2*∆*elp6*∆ cells. We isolated monomeric 80S ribosomes using a sucrose gradient after mild nuclease digestion and collected two independent datasets for each of the strains (Supplementary Table S1). From these images, we were able to reconstruct structures of translating ribosomes with an overall resolution of up to 1.8 Å allowing us to distinguish six distinct elongation states (Supplementary Fig. S2A-E). However, we did not observe major changes in the distribution of elongation states between the two strains, except for a marginal enrichment of post-translocation (POST) states in *ncs2*∆*elp6*∆ yeast (Supplementary Fig. S2B). This suggests that the observed phenotypic changes are caused by defects in specific decoding events and that the lack of U_34_ modification does not generally trap translating ribosomes in a specific state.

### tRNA modifications are critical for the decoding of codon pairs

**Figure 2.**
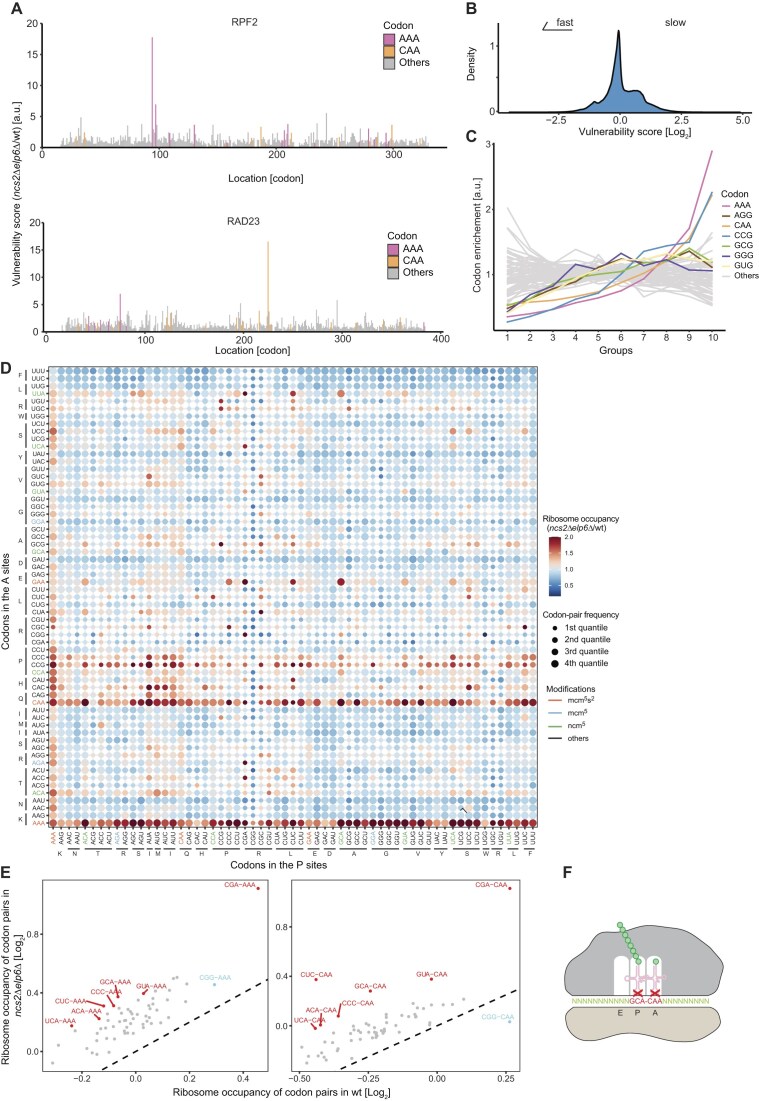
Strong pausing occurs at tRNA modification-dependent codon pairs. (**A**) The distribution of vulnerability scores for each codon in two example genes, *RPF2* and *RAD23*. Vulnerability scores are calculated by normalizing the pause score of codons in mutant yeast to those in wild type. AAA (magenta) and CAA (orange) are highlighted. A total of 15 codons at the start and end of the coding sequence are excluded from the calculation. (**B**) The distribution of vulnerability scores from all codons in the transcriptome. (**C**) Codons are equally divided into 10 groups according to the rank of vulnerability scores. The enrichment of each codon in each group is calculated. U_34_-modification-dependent codons (according to Fig. 1D; marked by colors) are enriched in the groups with higher vulnerability scores. (**D**) Two-dimensional codon plot depicting the change of ribosome occupancy for codon pairs between the *ncs2*∆*elp6*∆ mutant and wild type. Three biological replicates were used for both genotypes. Indicated on the x-axis are the codons in the ribosomal P site; on the y-axis are the codons in the ribosomal A site. Dark red color indicates a high ratio of ribosome occupancy in the *ncs2*∆*elp6*∆ mutant compared to wild type. For better visualization, ratios ≥2 were set to 2. Size of the dots represents the frequency of each codon pair in yeast categorized into four quantiles. Codons cognate to U_34_ modified tRNAs are indicated by color: mcm^5^s^2^U (red); mcm^5^U (blue); ncm^5^U (green). (**E**) Specific codon pairs are selected from panel (D). Ribosome occupancy between *ncs2*∆*elp6*∆ and wild-type yeast were compared for NNN–AAA (left) and NNN–CAA (right) codon pairs. The diagonal line indicates no change between mutant and wild type. Codon pairs that are more affected than the other pairs are highlighted in red. CGG–AAA and CGG–CAA (highlighted in blue) were weakly or not affected, and CGG–CAA was used as a negative control in Fig. 3. (**F**) Pausing ribosome translating a pair of U_34_-modification-dependent codons in the P and A sites, such as GCA–CAA.

The translation speed of codons within the same transcript can vary substantially [42, 69, 70]. However, identical codon optimality is generally assigned to codons regardless of their position or context in the transcriptome. The high resolution of our data allowed us to ask which codons in the transcriptome are most affected in the modification mutants and to investigate whether the sequence context affects the optimality of individual instances of a codon. Therefore, we computed a vulnerability score for all codons in the transcriptome by calculating the ratio of pause scores between *ncs2*∆*elp6*∆ mutants and wild-type yeast. This score provides a quantitative measure on how sensitive a particular codon is to the absence of these tRNA modifications (see the ‘Materials and methods’ section). Surprisingly, while AAA and CAA codons are on average decoded more slowly in *ncs2*Δ*elp6*Δ cells (Fig. 1D and Supplementary Fig. S1C), the extent of the slowdown varies substantially for different instances of these codons, even within the same transcript (Fig. 2A). Therefore, we ranked all codons in the transcriptome based on their vulnerability scores and divided them into 10 equally sized bins from low to high vulnerability (Fig. 2B). Strikingly, codons that depend on U_34_ modifications (AAA, CAA, AGG, GGG, CCG, GCG, and GUG) are more prevalent in bins with high vulnerability compared to bins with low vulnerability (Fig. 2C). Meanwhile, codons unrelated to modifications are distributed evenly. This demonstrates that our method effectively identifies and ranks codons according to their sensitivity to the absence of U_34_ modifications. When comparing the pause scores between mutant and wild-type samples, only 11% (CAA) and 14% (AAA and CCG) of codons are significantly slower than the average codons of the same instance in mutants. For unrelated codons, this value is roughly 3.5% (Supplementary Fig. S3A). These results suggest that the phenotypes observed in modification mutants are triggered by only a subset of all modification-dependent codons.

It is unexpected that only a small number of codons within the transcriptome respond to the absence of tRNA modifications while most instances of these codons do not. This finding immediately raises the question of what distinguishes vulnerable codons from those that are not affected. Therefore, we selected highly vulnerable codons and searched for features explaining why they are more affected by a lack of tRNA modifications than less vulnerable codons. Several studies have indicated that the region close to the 5′ end of transcripts is enriched with nonoptimal codons [71, 72]. However, we did not observe an enrichment of vulnerable codons in specific regions of the ORF, including the 5′ end (Fig. 2A and Supplementary Fig. S3B). Instead, we found that codons encoding the negatively charged amino acids aspartic acid and glutamic acid are strikingly depleted in the P site of vulnerable AAA, CAA, and GAA codons, but not upstream of U_34_-independent codons, such as UAU (Supplementary Fig. S3C). This suggests that electrostatic interactions of amino acids in the P site reduce the need of U_34_ tRNA modifications in A-site decoding.

The amino acids enriched in the P site do not share chemical properties and are unlikely to explain codon vulnerability. Therefore, we asked whether the extent of slowdown is modulated by the codon context. To visualize synergistic effects of adjacent codons in the *ncs2*∆*elp6*∆ mutant, we generated a two-dimensional plot depicting the change of translation speed at the P–A sites compared to wild type (Fig. 2D). As expected, AAA and CAA as well as other U_34_-dependent codons are overall slow during A-site decoding, regardless of the P-site codon. This is indicated by the horizontal red lines in the plot. Interestingly, we observed several strong stalling codon pairs when CGA is in the P site and a codon decoded by an U_34_-modified tRNA is in the A site. Examples include CGA–AGA, and CGA–CAA (Supplementary Fig. S3D). This is consistent with the observation that P–A codon pairs affect translation efficiency [11, 73] and suggests that the presence of CGA in the P site enhances dependence on U_34_ modifications for decoding A-site codons, potentially by altering the secondary structure of the mRNA around the decoding center [73]. Surprisingly, codons cognate to ncm^5^U-modified tRNAs (ACA, GCA, GUA, and UCA) at the P site also slow down the decoding of mcm^5^s^2^U-dependent codons (AAA, CAA, and GAA) at the A site in *ncs2*Δ*elp6*Δ yeast (Fig. 2E and F and Supplementary Fig. S3D–F). However, these ncm^5^U-dependent codons are decoded at normal speeds in the ribosomal A site (Fig. 1D and Supplementary Fig. S1C). These data demonstrate that modified tRNAs in the P site synergistically increase the optimality of A-site codons, and that codon vulnerability is largely explained by codon pairs. Finally, U_34_ modifications play strikingly different roles at the A and P sites. A lack of ncm^5^U affects wobble decoding at the A site, whereas ncm^5^U deficiency impairs cognate codon-anticodon pairing at the P site. In conclusion, U_34_ modifications on tRNAs at both ribosomal sites are critical for tuning translation elongation.

## tRNA modification defects induce ribosome collision

**Figure 3.**
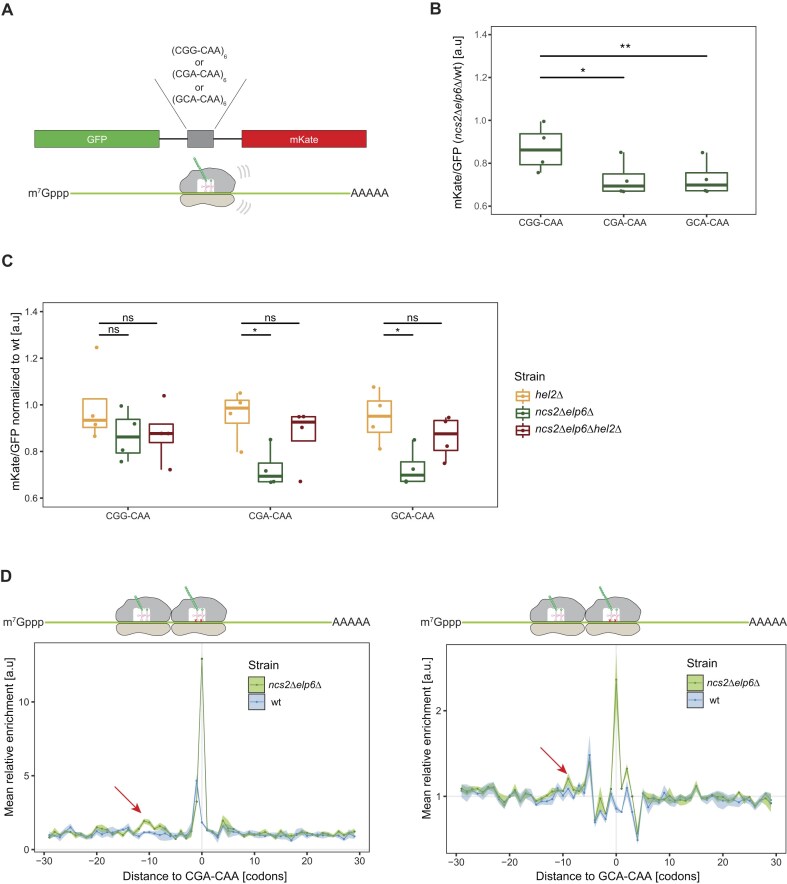
Ribosomes stall on slow codon pairs. (**A**) Schematic of the stalling reporter: six copies of CGA–CAA (slow), GCA–CAA (slow), or CGG–CAA (fast) were inserted between GFP and mKate. (**B**) The mKate/GFP ratios of CGG–CAA, CGA–CAA, and GCA–CAA reporters in *ncs2*Δ*elp6*Δ strains were normalized to wild type. Both stalling pairs show a decreased ratio in comparison to the control pair (two-sided Student’s *t*-test; *P-value <0.05, **P-value <0.01). (**C**) Similar to panel (B) for *hel2*∆, *ncs2*∆*elp6*∆ and *ncs2*∆*elp6*∆*hel2*∆ mutants. Knocking out *HEL2* in addition to *NCS2* and *ELP6* rescues the stalling effects of codon pairs. A *HEL2* knockout was used as a control (two-sided Student’s *t*-test; *P-value <0.05). (**D**) The distribution of ribosome occupancy (monosomes) around CGA–CAA (left) and GCA–CAA (right). Three biological replicates were used for both genotypes. Colliding ribosomes ∼10 codons upstream of the codon pairs in the *ncs2*∆*elp6*∆ mutant are highlighted by red arrows. The shaded area indicates the degree of experimental variation. The cartoon above depicts collided ribosomes relative to the plot.

To further substantiate the hypothesis that loss of tRNA modifications causes ribosomal stalling at U_34_-dependent codon pairs, we generated reporters containing six consecutive codon pairs between GFP and mKate (Fig. 3A) [74]. We combined the slow mcm^5^s^2^U-dependent codon CAA with either CGA or the ncm^5^U-dependent GCA (Fig. 3A). As a negative control, we used the fast CGG–CAA pairs (Figs 2E and 3A). When ribosomes stall during translation of nonoptimal codon pairs, less mKate is synthesized, as indicated by a low mKate/GFP ratio. Indeed, both slow codon pairs exhibited a ∼20% decrease in the mKate/GFP ratio in the *ncs2*∆*elp6*∆ mutant relative to the wild type (Fig. 3B), suggesting that ribosomes stall at U_34_-dependent nonoptimal codon pairs. Strong stalling induces ribosomal collisions and triggers the RQC pathway [39]. We therefore asked whether RQC is triggered in *ncs2*∆*elp6*∆ cells expressing stalling reporters and analyzed *ncs2*∆*elp6*∆*hel2*∆ cells, which lack Hel2, the E3 ubiquitin ligase that senses collided ribosomes upstream of the RQC pathway [35, 39, 41, 75]. Deleting *HEL2* indeed increases the mKate/GFP ratio for the nonoptimal CGA–CAA and GCA–CAA codon pairs but not for the CGG–CAA control (Fig. 3C), confirming that RQC is activated at U_34_ modification-dependent codon pairs in the absence of the modifications.

To verify that ribosomes stall similarly on endogenous transcripts, we searched our ribosome profiling data for evidence of colliding ribosomes, which are characterized by a peak in ribosome occupancy 10 codons upstream of the A site of stalled ribosomes [76]. Interestingly, we observed such a peak ∼10 codons upstream of CGA–CAA (Fig. 3D, left), GCA–CAA (Fig. 3D, right), and CGA–AAA pairs (Supplementary Fig. S4A). However, a similar peak was not found upstream of single AAA or CAA codons (Supplementary Fig. S4B), indicating that ribosome collisions are induced by stalling at tRNA modification-dependent codon pairs rather than single codons. When recognizing collided ribosomes, Hel2 ubiquitinates uS10 and synthesizes ubiquitin chains on uS3 at specific lysine residues, while Mag2 detects slow ribosomes independent of collisions [77–79]. To test whether RQC is active in U_34_-modification mutants, we assessed the ubiquitination of disomes from *ncs2∆elp6∆* and *ncs2*∆*elp6*∆*hel2*∆ cells by mass spectrometry. We observed K6/8 ubiquitination of uS10 in *ncs2*∆*elp6*∆ cells but it was not detectable on ribosomes from *ncs2*∆*elp6*∆*hel2*∆ cells (Supplementary Fig. S5A). Interestingly, uS3 was ubiquitinated at K212 in *ncs2*∆*elp6*∆ cells, while K212 ubiquitination levels of uS3 were reduced in *ncs2*∆*elp6*∆*hel2*∆ cells (Supplementary Fig. S5A). Taken together, our results provide strong evidence that specific U_34_-dependent codon pairs cause severe ribosomal stalling in tRNA-modification mutants *in vivo*, leading to ribosome collisions that trigger the RQC pathway via Hel2.

## The RQC and ISR pathways remedy codon-specific translation defects

**Figure 4.**
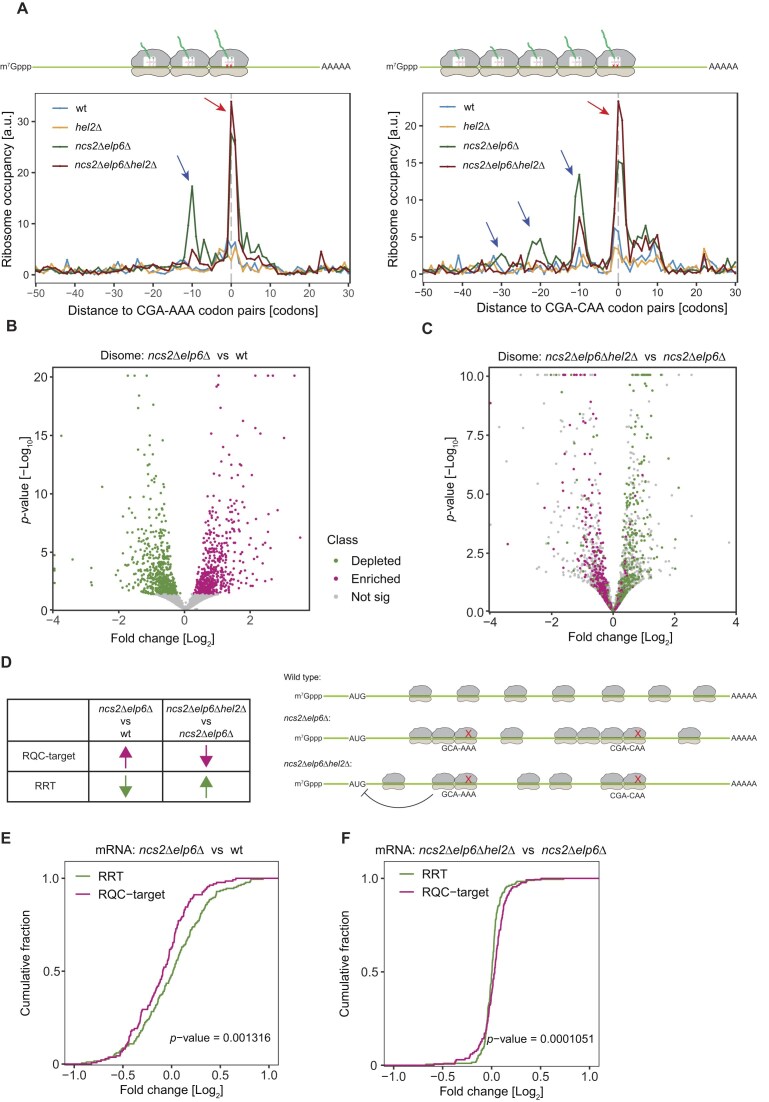
Ribosomes collide and are targeted by RQC. (**A**) Disome occupancy around CGA–AAA (left) and CGA–CAA (right) codon pairs in wild-type, *hel2*∆, *ncs2*∆*elp6*∆, and *ncs2*∆*elp6*∆*hel2*∆ yeast. The average disome occupancy from two biological replicates was plotted. Peaks at position 0 represent the A site of the first stalling ribosome (red arrow). One queueing disome (blue arrow) was observed upstream of CGA–AA codon pairs at position −10 because the size of ribosome footprints is ∼10 codons (left). This indicates three ribosomes at the stalling site. Upstream of CGA–CAA codon pairs, three queueing disomes were observed at position −10, −20, and −30 in *ncs2*∆*elp6*∆, but only one queueing disome was found at position −10 in *ncs2*∆*elp6*∆*hel2*∆. The cartoon above depicts collided ribosomes relative to the plot. (**B**) Differential expression of disome footprints comparing *ncs2∆elp6∆* to wild type and comparing *ncs2*∆*elp6*∆*hel2*∆ to *ncs2*∆*elp6*∆ using DESeq2 [58]. Monosome footprints were used in the differential expression analysis for normalization due to a positive correlation between disome and monosome footprints (see the ‘Materials and methods’ section). Genes that show disome enrichment in *ncs2*∆*elp6*∆ are highlighted in magenta while genes with less disomes are highlighted in green. (**C**) Disome-enriched or -depleted genes in panel (B) are highlighted in the differential-expression analysis between *ncs2*∆*elp6*∆*hel2*∆ and *ncs2*∆*elp6*∆. (**D**) The strategy to assign genes as an RQC target (*n* = 137) according to the disome enrichment in panel (B) and depletion in panel (C) or vice versa for RRTs (*n* = 188). Only genes significant (adjusted *P*-value <0.05) for both criteria were selected. The schematic diagram on the right panel depicts the enrichment or depletion of ribosomes on mRNAs in three strains. (**E**) The change of mRNA levels comparing *ncs2*∆*elp6*∆ to wild type was analyzed using DESeq2 [58]. (**F**) Similar to panel (E) but comparing *ncs2*∆*elp6*∆*hel2*∆ to *ncs2*∆*elp6*∆. The log_2_ fold change of RQC targets and RQC-refractory transcripts (RRTs) was compared (one-sided Kolmogorov–Smirnov test).

Collided ribosomes can be specifically studied by extracting disomes instead of monosomes during ribosome profiling [44, 80–83]. Therefore, we generated disome libraries of *hel2*∆, *ncs2*∆*elp6*∆, *ncs2*∆*elp6*∆*hel2*∆, and wild-type yeast. We observed pronounced ribosome collisions upstream of the strong stalling pairs CGA–AAA, CGA–CAA, GCA–AAA, and GCA–CAA in *ncs2∆elp6*∆ cells (Fig. 4A and Supplementary Fig. S5B). Importantly, the extent of stalling was further increased in the *ncs2*∆*elp6*∆*hel2*∆ mutant (Fig. 4A and Supplementary Fig. S5B; red arrow), but not at sites of CGG–AAA or CGG–CAA (Supplementary Fig. S5C), confirming that RQC resolves ribosome collisions at strong stalling sites in *ncs2∆elp6*∆ cells.

Furthermore, we observed a strong signal of a third ribosome colliding with the disome for CGA–AAA, GCA–AAA, and even longer ribosomal queues for CGA–CAA (Fig. 4A and Supplementary Fig. S5B; blue arrows) in the *ncs2*∆*elp6*∆ mutant. Interestingly, when *HEL2* is knocked out in *ncs2*∆*elp6*∆ cells, ribosome queueing is reduced compared to the *ncs2*∆*elp6*∆ mutant (Fig. 4A), indicating that ribosomal queueing is alleviated when the RQC pathway is inactivated. We posit that this effect is likely due to a downregulation of translation initiation, as we observed reduced rates of protein synthesis in *ncs2*∆*elp6*∆*hel2*∆ cells compared to the wild type and *ncs2*∆*elp6*∆ mutants (Supplementary Fig. S5D). Furthermore, and consistent with previous findings [37, 44], inactivation of RQC leads to activation of the ISR via Gcn2, resulting in increased translation of the transcription factor Gcn4 (Supplementary Fig. S5E) and transcriptional upregulation of its target genes (Supplementary Fig. S5F). In the absence of RQC, reduced translation initiation rates decrease the number of ribosomes on the transcript and reduce the likelihood of ribosome collisions.

Hel2-dependent ubiquitination marks ribosomes for splitting and the subsequent degradation of affected mRNA and nascent chains [36]. Since disomes indicate ribosome collisions, we compared their levels in wild-type, *ncs2*∆*elp6*∆, and *ncs2*∆*elp6*∆*hel2*∆ cells to identify transcripts targeted by the RQC pathway in U_34_-modification mutants. We identified transcripts that showed an increase in disomes in the *ncs2*∆*elp6*∆ mutant compared to wild type (Fig. 4B). These transcripts were enriched for disomes in the U_34_-modification mutant and were rescued in RQC-deficient *ncs2*∆*elp6*∆*hel2*∆ cells (Fig. 4C). This finding is consistent with the idea that there is reduced ribosomal queueing upstream of codon pairs in the *ncs2*∆*elp6*∆*hel2*∆ mutant (Fig. 4A). Our analysis enabled us to identify endogenous RQC targets in a genome-wide manner. We selected transcripts that were significantly upregulated in *ncs2*∆*elp6*∆ yeast but significantly downregulated in *ncs2*∆*elp6*∆*hel2*∆ cells as RQC target transcripts (Fig. 4B and C). We considered transcripts with the opposite pattern (downregulated in *ncs2*∆*elp6*∆ and upregulated in *ncs2*∆*elp6*∆*hel2*∆) to be RQC-refractory transcripts (RRTs) and used them as a negative control (Fig. 4D). In total, we identified 188 RQC target transcripts and 137 RRTs (Supplementary Table S2). Using these two groups, we determined the frequency of modification-sensitive codons in each category. RQC targets are significantly enriched for nonoptimal codons and codon pairs that depend on U_34_ modifications compared to RRTs (Supplementary Fig. S5G). However, whereas RRT are enriched for genes implicated in general metabolic processes such as amino acid biosynthesis or nucleoside metabolism (Supplementary Fig. S5H, left), RQC-targets are only weakly linked to functional categories in GO analyses (Supplementary Fig. S5H, right) suggesting that tRNA modifications are not used to functionally group transcripts in yeast.

It is thought that mRNAs recognized by the RQC pathway are degraded by NGD [32, 33]. Therefore, we investigated whether NGD-mediated degradation of RQC target transcripts occurs in tRNA modification mutants. We found that mRNA levels of RQC targets are significantly lower than those of RRTs in *ncs2*∆*elp6*∆ cells, but that this effect was reversed in the *ncs2*∆*elp6*∆*hel2*∆ mutant (Fig. 4E and F). To rule out the possibility of an indirect effect mediated by mRNA transcription, we generated 5PSeq datasets to quantify actively degrading mRNA (Supplementary Fig. S5I and J) [84]. Importantly, we observed increased degradation of RQC targets in *ncs2*∆*elp6*∆ cells; however, this effect was abolished in *ncs2*∆*elp6*∆*hel2*∆ cells (Supplementary Fig. S5I), demonstrating that the levels of RQC target mRNA are rescued in the absence of Hel2. We also observed a striking signal of degradation upstream of ribosomes decoding CGA–AAA codons (Supplementary Fig. S5J; red arrow). However, the resolution of the library did not allow us to draw further codon-specific conclusions. Taken together, these findings demonstrate that defects in U_34_ modification slow down ribosomes at modification-dependent codon pairs, trigger ribosome collisions, and activate the RQC pathway, which leads to the degradation of the affected mRNA.

## The RQC pathway prevents U_34_-modification-dependent protein aggregation

**Figure 5.**
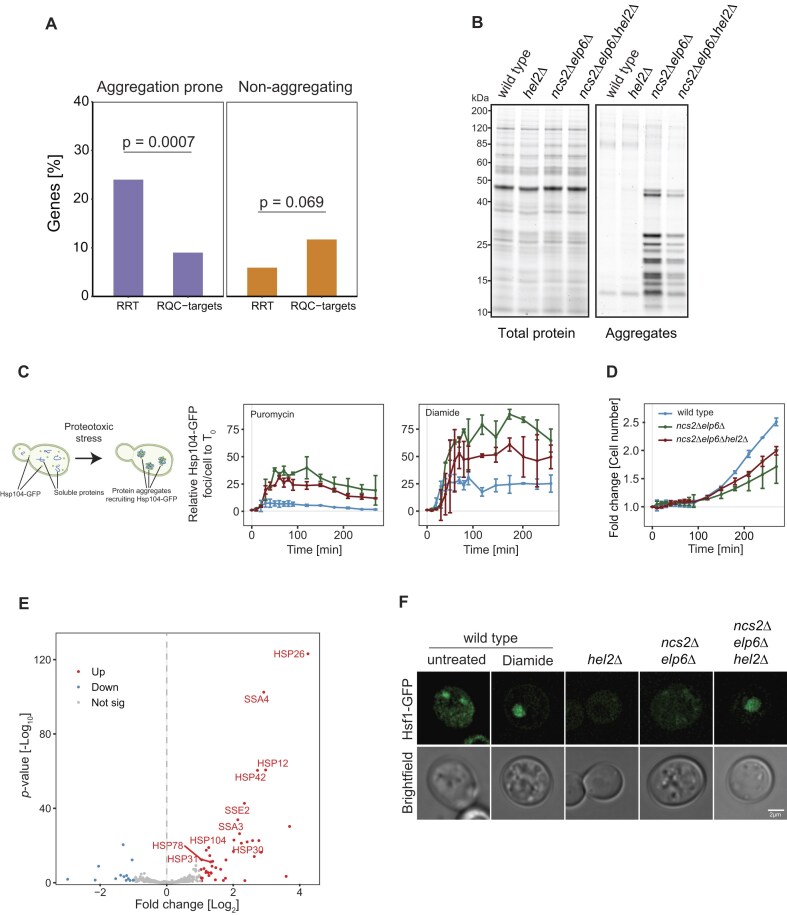
The RQC pathway alleviates protein homeostasis defects in coordination with chaperones. (**A**) Percentage of aggregation-prone (left) or non-aggregating (right) proteins in the RQC targets and RRTs identified in Fig. 5 (Fisher’s exact test). Proteins are considered as aggregation-prone in the mutant if enriched >2-fold in the aggregates compared to wild type. (**B**) Total protein (left) and aggregates (right) in wild-type, *hel2*∆, *ncs2*∆*elp6*∆, and *ncs2*∆*elp6*∆*hel2*∆ yeast. (**C**) Yeast cells expressing Hsp104-GFP as readout for protein aggregation were exposed to 10 mM puromycin (left), 2 mM diamide (right), and the formation of GFP-positive foci was quantified over 4 h. (**D**) Cell growth is monitored in Hsp104-GFP-based wild-type, *ncs2*∆*elp6*∆ and *ncs2*∆*elp6*∆*hel2*∆ strains. (**E**) Differential expression analysis of mRNA levels comparing *ncs2*∆*elp6*∆*hel2*∆ to *ncs2*∆*elp6*∆. Three biological replicates were used for *ncs2*∆*elp6*∆ and two biological replicates for *ncs2*∆*elp6*∆*hel2*∆. Upregulated genes with *P*-adjusted value <0.05 and log_2_ fold change >1 are highlighted in red while downregulated genes with *P*-adjusted value <0.05 and log_2_ fold change <−1 are highlighted in blue. Significantly upregulated chaperones are labeled. (**F**) Hsf1-GFP was used to detect the localization of Hsf1 by live-cell microscopy (top row: GFP; bottom row: brightfield; scale bar for all images in this panel: 2 µm). As positive control, wild-type cells were treated with 2 mM diamide.

A lack of U_34_ modifications triggers protein aggregation [10, 31, 85]. In light of our findings above, we asked how the observed ribosome collisions, RQC activation, and resultant mRNA degradation affect protein aggregation. Therefore, we reanalyzed a census of proteins that aggregate in the absence of U_34_-modifications [10]. Of the 1080 proteins detected by mass spectrometry in *ncs2*∆*elp6*∆ and wild-type yeast, we found that 610 proteins are more likely to aggregate in *ncs2*∆*elp6∆* cells compared to wild type, while 401 proteins are less likely to aggregate in the mutant, which we used as non-aggregating controls (see the ‘Materials and methods’ section; Supplementary Table S3). Interestingly, we found that aggregation-prone proteins are significantly enriched in RRTs compared to RQC targets, while we observed the opposite trend for non-aggregating proteins (Fig. 5A). This indicates that mRNA of non-aggregating proteins are more likely RQC target transcripts than mRNA of aggregation-prone proteins. Consistently, we detected more disomes in transcripts of non-aggregating proteins when comparing *ncs2*∆*elp6*∆ to wild-type cells, but reduced levels of disomes on the same transcripts in *ncs2*∆*elp6*∆*hel2*∆ cells lacking RQC activity (Supplementary Fig. S6A). Furthermore, mRNA levels of non-aggregating proteins were reduced in *ncs2*∆*elp6*∆ cells compared to the wild type and this effect was rescued in *ncs2*∆*elp6*∆*hel2*∆ cells (Supplementary Fig. S6B), indicating that RQC targets are less likely to form protein aggregates. Importantly, although we did not detect an enrichment of single U_34_-modification-dependent codons in either aggregation-prone or non-aggregating proteins (Supplementary Fig. S6C, left), we found a significant enrichment of U_34_-modification-sensitive codon pairs in non-aggregating proteins (Supplementary Fig. S6C, right). Since nonoptimal codon pairs are more likely to cause ribosome collisions than single codons (Fig. 3D and Supplementary Fig. S4A and B), the enrichment of such codon pairs in non-aggregating proteins further supports our finding that RQC targets are non-aggregating protein transcripts. Taken together, this suggests that the role of the RQC pathway extends beyond quality control of individual defective transcripts, but that it plays an additional role in preventing aggregate formation when translation dynamics are suboptimal. By degrading transcripts that accumulate collided ribosomes and potentially dysfunctional peptides, the RQC pathway reduces protein aggregation.

## The RQC pathway is coordinated with molecular chaperones

To understand the impact of RQC on protein homeostasis, we next asked how the absence of Hel2 affects protein aggregation in cells lacking U_34_ tRNA modifications. Therefore, we purified endogenous protein aggregates from wild-type, *hel2*∆, *ncs2*∆*elp6*∆, and *ncs2*∆*elp6*∆*hel2*∆ cells [10, 30]. We expected to observe an increase in protein aggregates in *ncs2*∆*elp6*∆*hel2*∆ cells due to the accumulation of defective mRNAs and misfolded proteins. However, we found that the levels of aggregating proteins were lower in *ncs2*∆*elp6*∆*hel2*∆ cells compared to *ncs2*∆*elp6*∆ yeast (Fig. 5B). To confirm this finding, we performed high-content microscopy using Hsp104-GFP to monitor the formation of protein aggregates in response to chemically perturbed translation or protein stress *in vivo*. Knocking out *HEL2* reduced the formation of Hsp104-GFP foci in *ncs2*∆*elp6*∆ cells exposed to stress-inducing drugs (Fig. 5C). Furthermore, removing Hel2 rescued the growth defect of U_34_-modification mutants (Fig. 5D). This suggests that an additional quality control system contributes to preventing protein aggregation in the absence of functional RQC.

It was previously shown that ISR may reduce protein aggregation in a manner similar to the triple knockout used in our study [37]. To further delineate the molecular mechanisms underlying this effect, we analyzed mRNA expression of *ncs2*∆*elp6*∆*hel2*∆ and *ncs2*∆*elp6*∆ yeast. This analysis revealed a striking increase in the expression of molecular chaperones in *ncs2*∆*elp6*∆*hel2*∆ cells (Fig. 5E) and gene ontology (GO) terms associated with protein folding (Supplementary Fig. S6D). However, chaperone expression was not altered in *ncs2*∆*elp6*∆ or *hel2*∆ cells compared to the wild type (Supplementary Fig. S6E and F). These results suggest that the RQC pathway monitors suboptimal translation dynamics in a manner antagonistic to molecular chaperones to maintain cellular protein homeostasis. Heat shock factor 1 (Hsf1) is a key player that integrates the cellular response to numerous physiological stresses [86]. Downstream of the RQC pathway, Hsf1 receives a distinct stress signal via Tae2/Rqc2 [39]. Under normal conditions, Hsf1 is sequestered in the cytoplasm by molecular chaperones. However, when protein misfolding occurs, chaperones bind to their client proteins and release Hsf1. Hsf1 then translocates to the nucleus and upregulates chaperone expression [87]. Since Hsf1 localization indicates its chaperone-inducing activity, we monitored its localization and found that it is primarily cytoplasmic in *hel2*∆, *ncs2*∆*elp6*∆, and wild-type cells. However, Hsf1-GFP relocalizes to the nucleus in *ncs2*∆*elp6*∆*hel2*∆ cells, similar to the effect of diamide treatment, which was used as a positive control (Fig. 5F). These findings demonstrate that Hsf1 mediates the chaperone response despite the absence of RQC signaling. This implies that the RQC pathway is generally sufficient to prevent the heat-shock response. In summary, we conclude that the heat-shock response acts as a secondary cellular response that is activated when the RQC pathway is inactive and unable to suppress protein homeostasis failure caused by codon-specific translation defects.

## Discussion

Codon-specific translation rates, and therefore codon optimality, are generally determined by averaging ribosome occupancy of all instances of a codon across the transcriptome [8–10, 88–90]. Here, we used high-resolution ribosome profiling to pinpoint translational slowdown events with high positional accuracy, revealing stark context-dependency to translational perturbations. Using yeast devoid of U_34_ tRNA modifications to probe the effects of codon-specific translational slowdown, we reveal a differential requirement for tRNA modifications between the ribosomal A site and P site, ribosome stalling at specific codon pairs that trigger ribosome collisions, and a hierarchical interplay between RQC and molecular chaperones to resolve translation impairments and protein homeostasis defects.

While previous low-resolution studies revealed slowdown during the decoding of AAA and CAA by their cognate mcm^5^s^2^U-modified ${\mathrm{tRNA}}_{{\mathrm{UUU}}}^{{\mathrm{Lys}}}$ and ${\mathrm{tRNA}}_{{\mathrm{UUG}}}^{{\mathrm{Gln}}}$ in cells lacking U_34_ modifications [9, 10], our high-resolution analysis uncovered that ncm^5^U and mcm^5^U are required during wobble decoding of a subset of G-ending codons (AGG, CCG, GCG, GGG, and GUG; Fig. 1D and Supplementary Fig. S1C) in the ribosomal A site. Unexpectedly, we also find that ncm^5^U is required for efficient processing of cognate codons (ACA, GCA, GUA, UCA) in the P site (Fig. 2D and E and Supplementary Fig. S3D and E). This demonstrates that the same modification can differentially tune the anticodon-codon interaction of different codons in the A and P site of the ribosome. To our knowledge, such an effect has not been reported previously.

Importantly, we observed that specific instances of codons are more vulnerable to modification defects compared to other codons of the same type, even within the same transcript. We systematically evaluated factors that explain the observed vulnerability and found that this phenomenon largely depends on the P-site codon that synergistically deoptimizes a suboptimal A-site codon (Fig. 2). Codon pair biases have been found in many organisms, where suboptimal codon pairs can compromise translation rates [38, 91, 92] or contribute to pausing, reduced gene expression and decreased mRNA stability [11, 93]. When analyzing slow codon pairs in U_34_-modification mutants, we found that U_34_-related codons and CGA in the P site specifically enhance the slowdown of A-site codons. CGA codons are commonly observed in slow pairs, which has been explained by a change in mRNA conformation in the P site [11, 38, 73]. Accordingly, a neural network model found a high predictive value of the P site for elongation speed in wild-type yeast [94]. We hypothesize that the absence of ncm^5^U or mcm^5^U during A-U pairing similarly affects mRNA structure at the P site, reducing the binding efficiency of hypomodified tRNA at the A site. Furthermore, our data strongly suggest that the phenotypes of tRNA modification mutants are mediated by defects in codon-pairs. Identifying codon pairs that become suboptimal under specific conditions is crucial because these cannot be predicted based on their frequency in the transcriptome and may differ when tRNA modification levels change in response to environmental conditions.

We demonstrate that ribosomes stall at suboptimal codon pairs, which leads to ribosome collisions (Figs 2, 3B, and 4A, and Supplementary Figs S3D, and E, S4A, and S5B). This triggers RQC, as indicated by Hel2-dependent ubiquitination of ribosomal protein uS10 (Supplementary Fig. S5A). Depletion of Hel2 increases disome occupancy at modification-dependent codon pairs in the absence of U_34_ modifications. This indicates that RQC resolves collided ribosomes at such stalling sites. However, in *ncs2*∆*elp6*∆*hel2*∆ cells, we observed reduced ribosome queueing upstream of CGA–CAA and CGA–AAA pairs, as well as fewer disome footprints at affected transcripts (Fig. 4A and Supplementary Fig. S5B). This counterintuitive finding suggests that a large fraction of mRNAs contain a single collision that is not resolved due to the inactivation of the RQC pathway, leading to disome accumulation at these sites. However, an alternative explanation is that translation initiation is inhibited, thereby reducing the ribosome load on mRNA and alleviating ribosomal queueing. Persistent ribosome collisions activate the *GCN2*-mediated stress response pathway, leading to a block in translation initiation [95]. Consistent with this, we observed reduced initiation in *ncs2*∆*elp6*∆*hel2*∆ cells (Supplementary Fig. S5D), as well as a striking upregulation of Gcn4 targets (Supplementary Fig. S5F), which is indicative of Gcn2 activation. In line with our results, it has been shown that RQC and ISR are antagonistic pathways that compete for collided ribosomes as their substrate [41, 96]. Therefore, deletion of *HEL2* promotes increased Gcn2 activation [37, 44].

**Figure 6.**
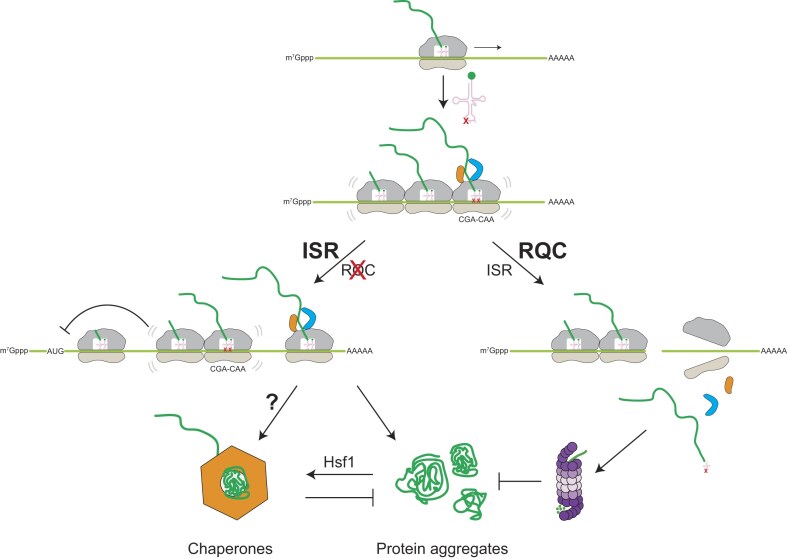
Working model. Model for the cooperative surveillance of the cellular quality-control systems in response to codon-specific translation defects induced by hypomodification of tRNA. When tRNA modifications are absent, ribosomes collide at modification-dependent codon pairs. This induces the RQC pathway, which targets the aberrant transcript (right). Peptides synthesized by stalling ribosomes are degraded by the proteosome and ribosome subunits recycled. Thus, RQC targets are less likely to form protein aggregates. When the RQC is inactive (left), the ISR is strongly induced and downregulates translation initiation to prevent further ribosome collisions, and the cell overexpresses chaperones via Hsf1 to reduce protein aggregation.

The combination of tRNA modification defects and RQC impairment induced a striking upregulation of molecular chaperones that we did not observe when either pathway was perturbed independently (Fig. 5E). This shows that the different quality control systems work hierarchically to ensure the synthesis of correctly folded proteins. Our study reveals that ribosomal stalling at slow codon pairs triggers the RQC pathway and that the nascent peptides of RQC target genes are likely degraded (Fig. 6). In addition to monitoring mRNA quality, we show a new role for RQC in preventing general protein aggregation by targeting suboptimal translation events at an early stage. If stalled ribosomes cannot be resolved by RQC, the defective nascent peptides have a high potential to interact with other proteins and form potentially harmful aggregates [97]. Importantly, the fact that chaperones are only slightly upregulated in *ncs2*∆*elp6*∆ yeast suggests that the chaperone response is suppressed when RQC is active [37, 39, 44]. It has been shown that the induction of Hsf1 through Tae2 differs from the induction by different stress pathways [39]. Our results provide further evidence for this. RQC and HSR likely act antagonistically to improve the cellular energy economy since chaperones require high levels of ATP. Under physiological conditions, RQC efficiently removes potentially misfolding proteins by simply degrading mRNA and the nascent chain, allowing subsequent recycling of the different components of the translation machinery. The role of RQC to recognize defects in translation dynamics and to avoid co-translational misfolding may become even more critical during the co-translational assembly of protein complexes by subsequent ribosomes [98, 99]. Although we have used yeast as a model system in this study, similar mechanisms likely occur in metazoans. For example, mutations of several tRNA modifying enzymes but also the RQC machinery are associated with neurodegenerative phenotypes [21, 22, 100]. Since neurons harbor complex proteostasis needs, coordination between quality-control pathways may contribute to the integrity of brain cells and prevention of neurodegeneration.

In conclusion, we show that U_34_ tRNA modifications differentially affect A-site and P-site codons, highlighting the importance of codon pairs for context-dependent codon optimality. Seemingly mild codon-specific translation defects can induce ribosome stalling and collisions through the synergistic effect of suboptimal codons. While RQC is able to correct mild translation defects, it is overwhelmed when such defects become persistent. When translation dynamics are perturbed, ribosomes deviate from the optimal balance between protein synthesis rates and cotranslational folding, forcing cells to remedy this perturbation by integrating multiple branches of cellular quality control. Although our study focused on U_34_ modifications, the conceptual framework can be extended to other factors that alter codon optimality, such as mutations in the translation machinery, tRNA copy number variations, or epitranscriptomic modifications [101, 102] . Therefore, the analysis of suboptimal codon pairs will likely be key for the design of expression constructs, including RNA therapeutics.

## Supplementary Material

gkaf1311_Supplemental_Files

## Data Availability

***Next generation sequencing:*** All data are available at the Gene Expression Omnibus (GEO); accession number: GSE245673. ***Proteomics:*** Data of disomes isolated from wild-type and *ncs2Δelp6Δ* ribosomes are available on the PRIDE database; accession number: PXD066757. ***Cryo-EM:*** The movie stacks and cryo-EM densities generated in this study have been validated at the Electron Microscopy Public Image Archive (EMPIAR) and the Electron Microscopy Data Bank (EMDB). Furthermore, micrographs and cryo-EM densities have been deposited under the following accession codes: (i) WT#1 PRE-H2 EMPIAR-12015, EMD-19947; (ii) WT#2 PRE-H2 EMPIAR-12016, EMD-19948; (iii) *ncs2Δelp6Δ*#1 PRE-H2 EMPIAR-12013, EMD-19945; (iv) *ncs2Δelp6Δ*#2 PRE-H2 EMPIAR-12014, EMD-19946; (v) PRE-NR EMD-19951; (vi) PRE-H1 EMD-19949; (vii) POST1-NR EMD-19952; (viii) POST2-NR EMD-19950; (ix) SC EMD-19953. ***Source code:*** The original code used for the analysis has been deposited at https://github.com/JieWu2012/U34_tRNA_modifications and is publicly available at https://zenodo.org/records/16227101.
