# Supplementary material for: Suboptimal codon pairs trigger ribosome collisions and cellular quality control responses in tRNA modification mutants
Source: Nucleic Acids Res. 2025 Dec 23;53(22):gkaf1311. doi: 10.1093/nar/gkaf1311 (PMC12721868; doi:10.1093/nar/gkaf1311)
Supplement: gkaf1311_Supplemental_Files [file gkaf1311_supplemental_files.zip › Wu_Eggers_Supp_Figures.pdf]

Suboptimal codon pairs trigger ribosome collisions and cellular quality control responses in tRNA modification mutants

Jie Wu[1,2,†], Cristian Eggers[1,2,†], Olga Sin[1], Łukasz Koziej[3,4], Hector Mancilla[5], Fabienne Mollet[1], Hans R. Schöler[6], Hannes C.A. Drexler[7], Tristan Ranff[8], Christian Fufezan[8], Claudine Kraft[5,9], Sebastian Glatt[3,4], Jan M. Bruder[6], Sebastian A. Leidel[1*]

[1]Department of Chemistry, Biochemistry and Pharmaceutical Sciences (DCBP), University of Bern, 3012 Bern, Switzerland.

[2]The Graduate School for Cellular and Biomedical Sciences (GCB), University of Bern, 3012 Bern, Switzerland.

[3]Malopolska Centre of Biotechnology (MCB), Jagiellonian University, 30387 Kraków, Poland

[4]Department for Biological Sciences and Pathobiology, University of Veterinary Medicine Vienna, 1210 Vienna, Austria.

[5]Institute of Biochemistry and Molecular Biology (ZBMZ), Faculty of Medicine, University of Freiburg, 79104 Freiburg, Germany.

[6]Department for Cell and Developmental Biology, Max Planck Institute for Molecular Biomedicine, 48149 Muenster, Germany.

[7]Bioanalytical Mass Spectrometry Unit, Max Planck Institute for Molecular Biomedicine, 48149 Muenster, Germany.

[8]Faculty of Engineering Sciences, Heidelberg University, 69120 Heidelberg, Germany

[9]Centre for Integrative Biological Signalling Studies (CIBSS), University of Freiburg, 79104 Freiburg, Germany.

*To whom correspondence should be addressed. Tel: +41 31 684 4296; Email: sebastian.leidel@unibe.ch

†The first two authors should be regarded as joint first authors.

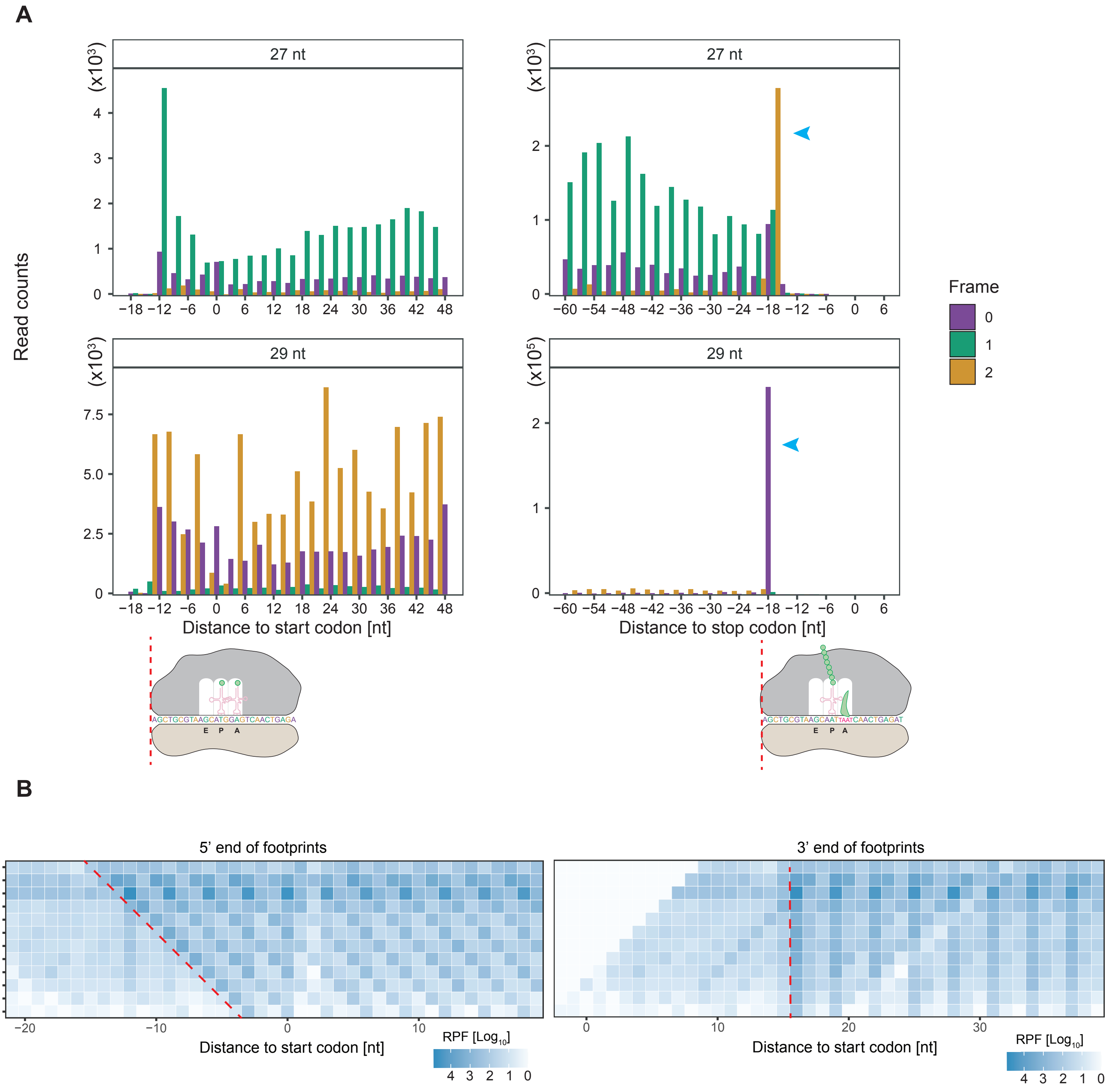

Supplementary Figure 1

C

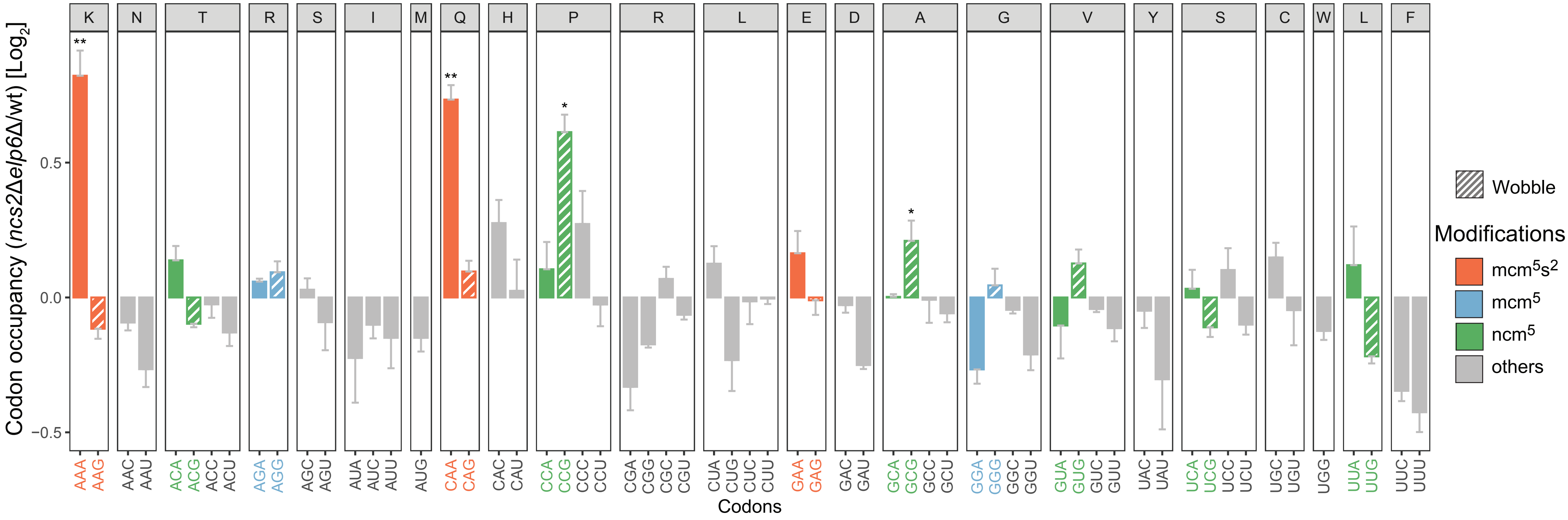

D

Codon	Anticodon	Amino acid	Codon	Anticodon	Amino acid	Codon	Anticodon	Amino acid	Codon	Anticodon	Amino acid
UUU	—	Phe (F)	UCU	IGA (11)	Ser (S)	UAU	—	Tyr (Y)	UGU	—	Cys (R)
UUC	GmAA (10)		UCC	—		UAC	GΨA (8)		UGC	GCA (4)	
UUA	ncm^{5}UmAA (7)	Leu (L)	UCA	ncm^{5}UGA (3)		UAA	—	n.a.	UGA	—	n.a.
UUG	m^{5}CAA (10)		UCG	CGA (1)		UAG	—		UGG	CmCA (6)	Trp (W)
CUU	—	Leu (L)	CCU	IGG (2)	Pro (P)	CAU	—	His (H)	CGU	ICG (6)	Arg (R)
CUC	GAG (1)		CCC	—		CAC	GUG (7)		CGC	—	
CUA	UAG (3)		CCA	ncm^{5}UGG (10)		CAA	mcm^{5}s^{2}UUG (9)	Gln (Q)	CGA	—	
CUG	—		CCG	—		CAG	CUG (1)		CGG	CCG (1)	
AUU	IAU (13)	Ile (I)	ACU	IGU (11)	Thr (T)	AAU	—	Asn (N)	AGU	—	Ser (S)
AUC	—		ACC	—		AAC	GUU (10)		AGC	GCU (4)	
AUA	ΨAΨ(2)		ACA	ncm^{5}UGU (4)		AAA	mcm^{5}s^{2}UUU (7)	Lys (K)	AGA	mcm^{5}UCU (11)	Arg (R)
AUG	CAU (5/5)	Met (M)	ACG	CGU (1)		AAG	CUU (14)		AGG	CCU (1)	
GUU	IAC (14)	Val (V)	GCU	IGC (11)	Ala (A)	GAU	—	Asp (D)	GGU	—	Gly (G)
GUC	—		GCC	—		GAC	GUC (16)		GGC	GCC (16)	
GUA	ncm^{5}UAC (2)		GCA	ncm^{5}UGC (5)		GAA	mcm^{5}s^{2}UUC (14)	Glu (E)	GGA	mcm^{5}UCC (3)	
GUG	CAC (2)		GCG	—		GAG	CUC (2)		GGG	CCC (2)	

Supplementary Figure 1

Supplementary Figure 1. U_{34} modifications affect translation differentially. (**A**) The distribution of the 5' ends of 27- (top) and 29-nt (middle) footprints is shown at the start (left) or at the stop (right) codon; (bottom) the cartoon depicts initiating and terminating ribosomes. The terminating ribosomes accommodate four nucleotides in the ribosomal A site due to eRF1 recognition, which is visible as an apparent frameshift at the stop codon (blue arrowhead). (**B**) The 5' end of initiating ribosome footprints shifts one nucleotide at a time with the length of footprints, while the 3' end of initiating ribosome footprints does not change with the length footprints. (**C**) Alterations in codon occupancy in the absence of U_{34} tRNA modification using a position-based normalization strategy (1). Even though AGG, GGG, and GUG do not score as significantly as in the read-based method (Figure 1D), they are slow compared to their synonymous A-ending codons. Three biological replicates were used for both genotypes. (**D**) Codon table including the tRNA anticodon with modifications. The numbers in brackets are the copy numbers of the tRNA genes in yeast. Known essential (blue) and non-essential (orange) tRNAs are marked with stars. Red dots connected by lines depict the modification-mediated wobble pairing discovered in this study. The codon table was adapted from Ref. (2).

A

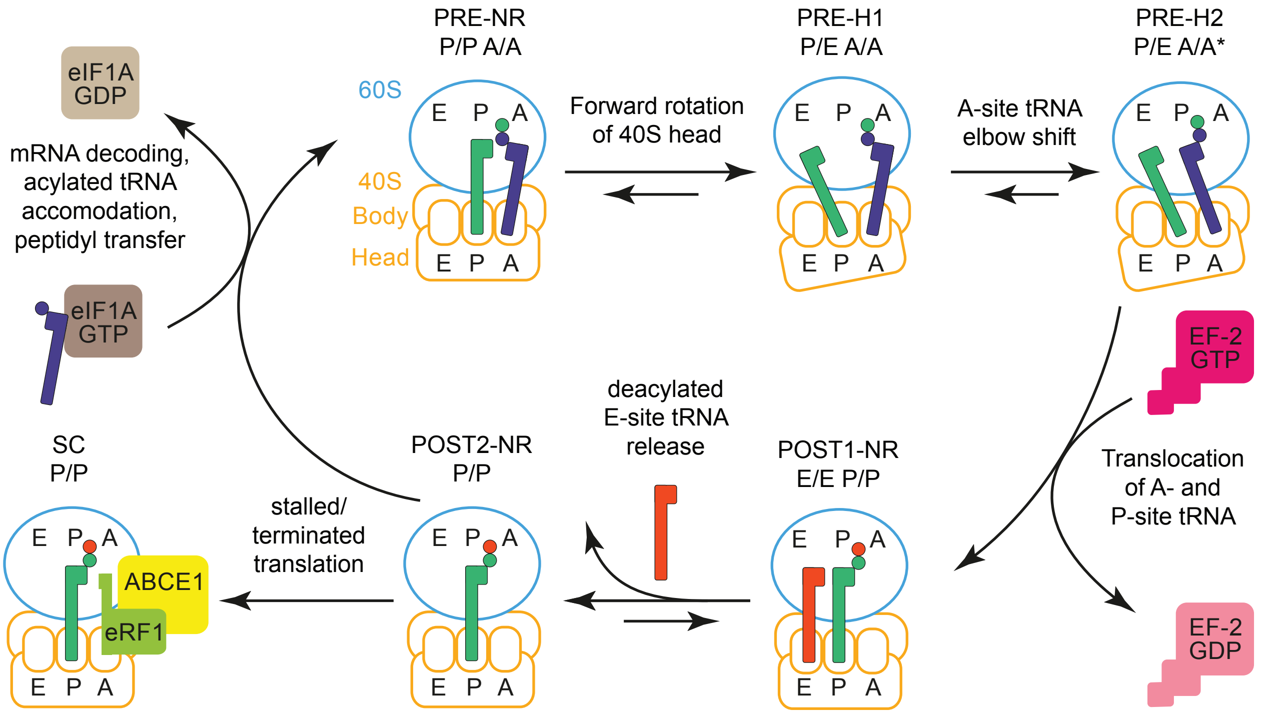

B

Dataset (#replicate)	PRE	POST	SC
WT#1	95.6%	4.2%	0.2%
WT#2	95.8%	4.0%	0.2%
ncs2Δelp6Δ#1	94.1%	5.6%	0.3%
ncs2Δelp6Δ#2	95.3%	4.5%	0.2%

particle distribution (%)

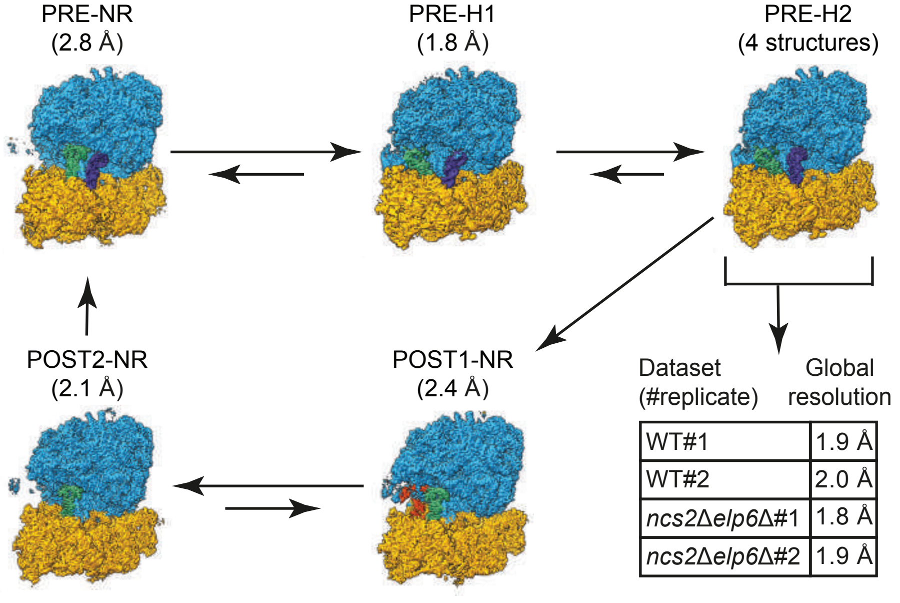

Dataset (#replicate)	Global resolution
WT#1	1.9 Å
WT#2	2.0 Å
ncs2Δelp6Δ#1	1.8 Å
ncs2Δelp6Δ#2	1.9 Å

Supplementary Figure 2

C

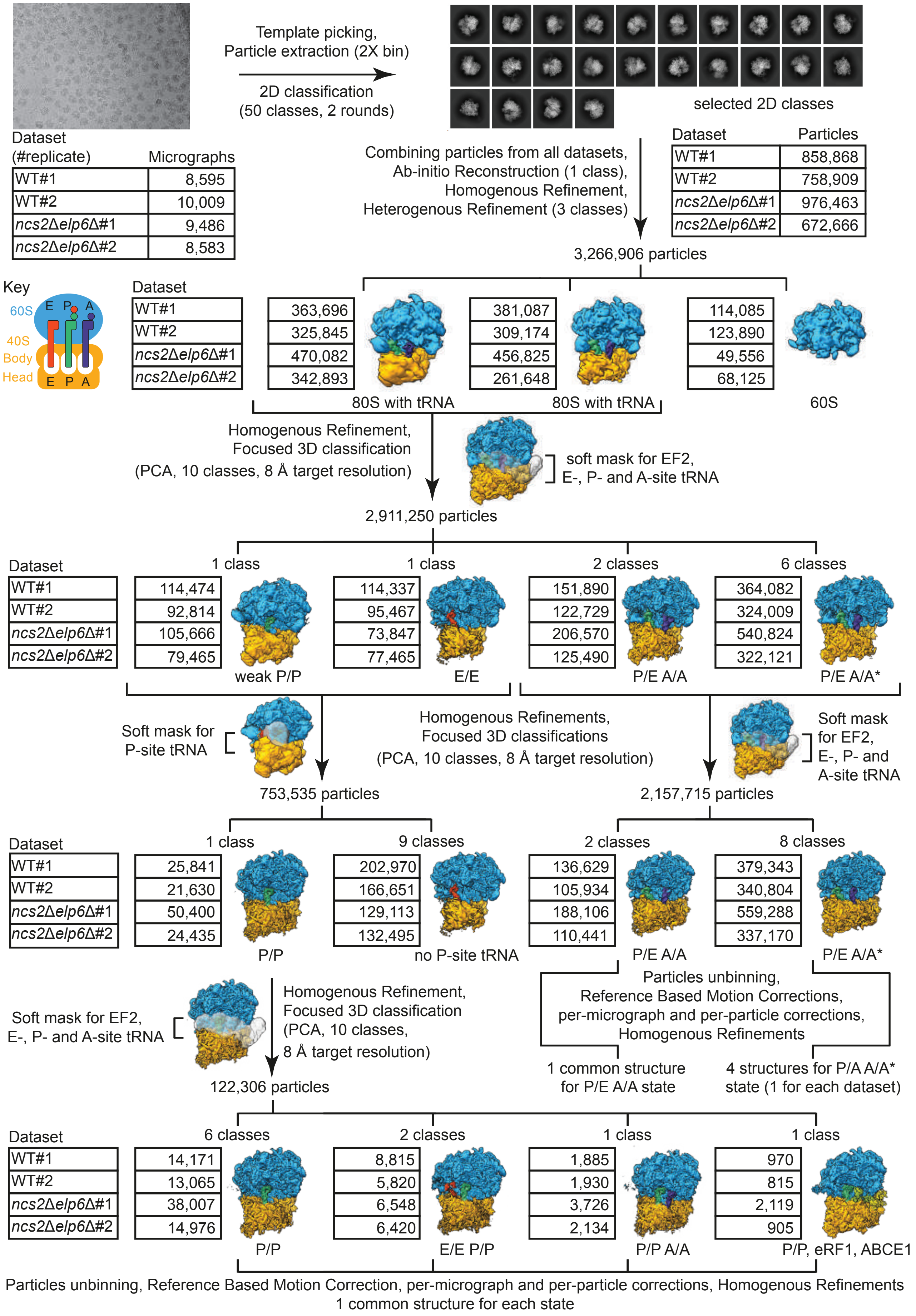

Supplementary Figure 2

D

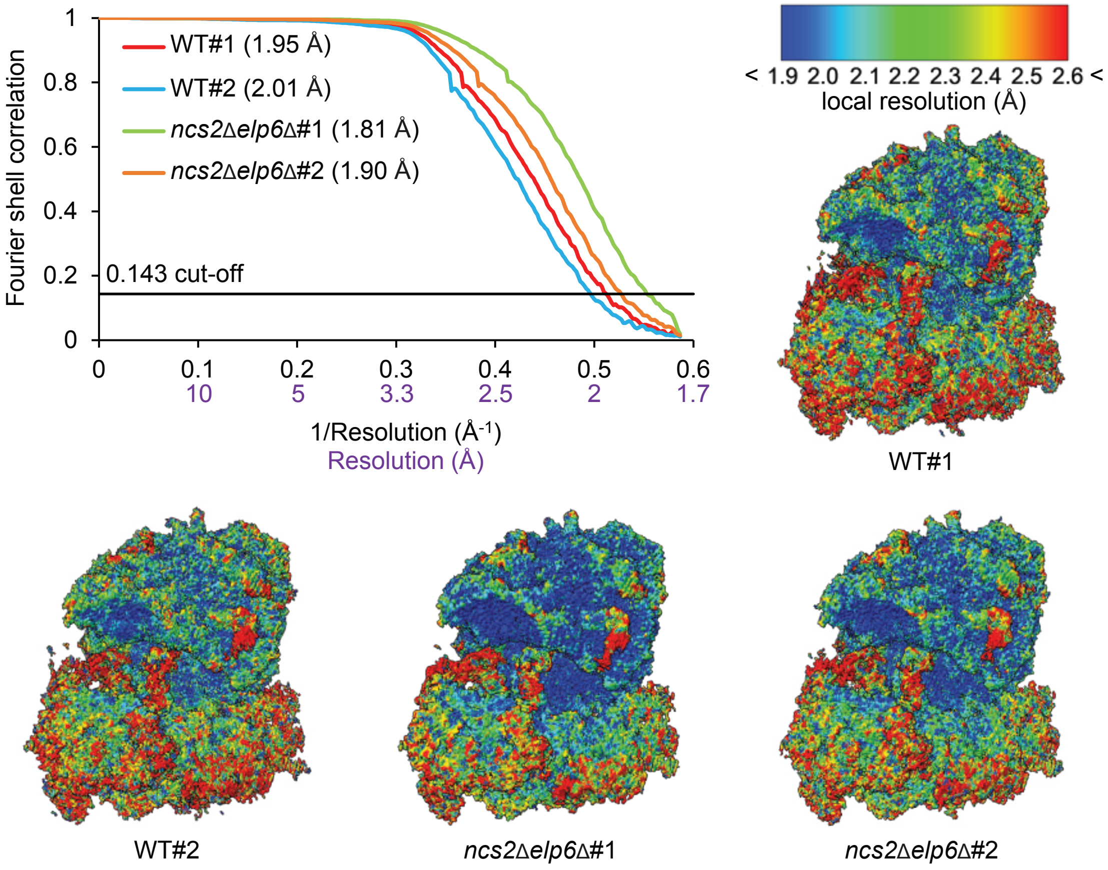

E

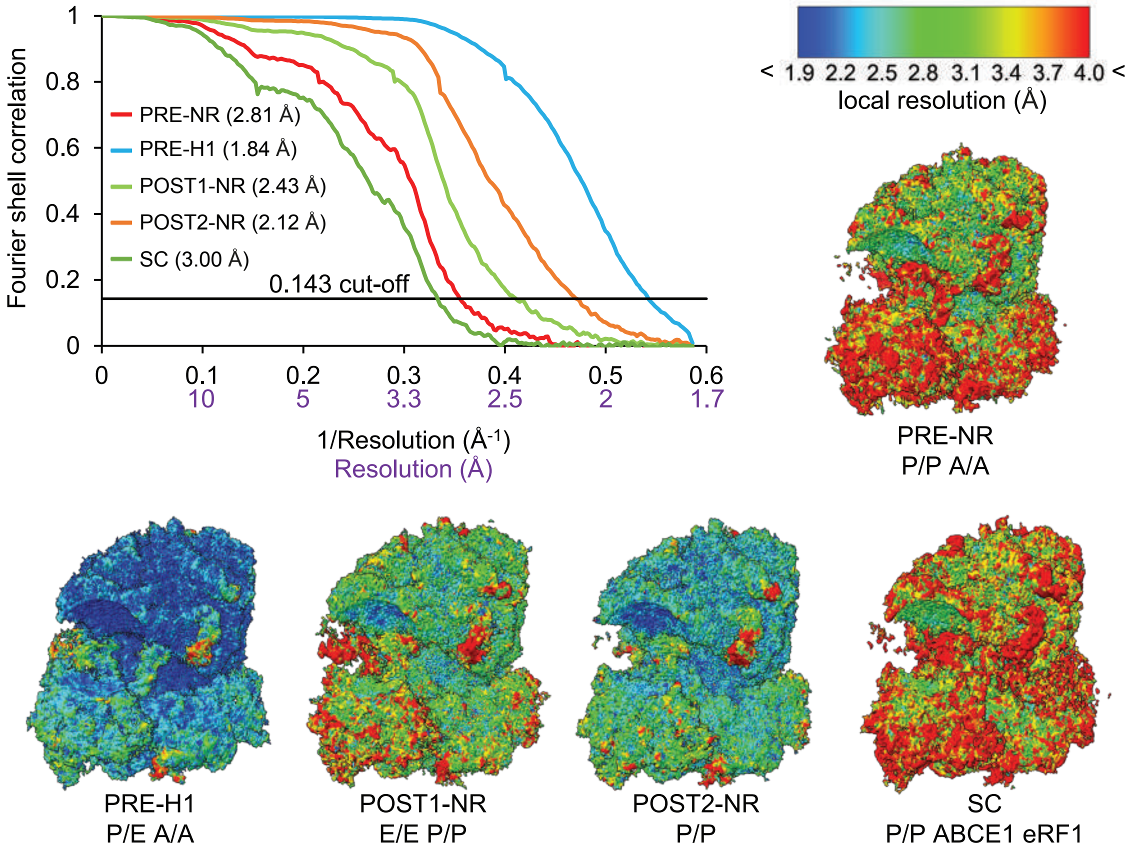

Supplementary Figure 2

Supplementary Figure 2. Cryo-EM analysis of wild-type and *ncs2Δelp6Δ* ribosomes. (**A-E**) Cryo-EM reconstructions of wild-type (WT) and *ncs2Δelp6Δ S. cerevisiae* translating ribosomes extracted by MNase digestion from polysomes. (**A**) Schematic depiction 80S ribosomes and relative positioning of 60S (blue) and 40S subunit (orange), E-, P-, and A-site tRNA (red, dark green, and purple) during translation elongation, before (PRE) and after translocation (POST). The forward and backward arrows indicate the transition and potential reversibility of intermediates. Six 80S translation elongation states reported in the current study are as follows: PRE-translocation non-rotated P/P A/A (PRE-NR); PRE-translocation-hybrid P/E A/A (PRE-H1); PRE-translocation-hybrid P/E A/A* (PRE-H2); post-translocation non-rotated E/E P/P (POST1-NR); post-translocation non-rotated P/P (POST2-NR); and splitting complex (SC) with P/P tRNA, eRF1 (light green), and ABCE1 (yellow). (**B**) Relative distribution (%) of 80S particles into PRE, POST and SC states and corresponding cryo-EM reconstructions. The cryo-EM datasets were collected in 2 replicates (#1/#2) for each WT and *ncs2Δelp6Δ S. cerevisiae* strain. Global resolutions (in Å) are reported for each cryo-EM structure (GSFSC with 0.143 cut-off). (**C**) Classification scheme of cryo-EM data for bulk 80S ribosomes from wild-type and *ncs2Δelp6Δ* yeast. The diagram depicts the strategy applied to remove junk particles by 2D classification and to separate the ribosomal particles into different states by Heterogenous Refinement and 3D classification. The Heterogenous Refinement allowed the removal of particles corresponding to the 60S subunit. The focused 3D classifications allowed the removal of the particles corresponding to non-translating 80S ribosomes (lacking P-site tRNA). Furthermore, the approach allowed separation and reconstruction of the following translationally-active 80S ribosomes: PRE-translocation non-rotated P/P A/A (PRE-NR); PRE-translocation-hybrid P/E A/A (PRE-H1); PRE-translocation-hybrid P/E A/A* (PRE-H2); post-translocation non-rotated E/E P/P (POST1-NR); post-translocation non-rotated P/P (POST2-NR); and splitting complex with P/P tRNA, eRF1, and ABCE1 (SC). (**D**) Global and local resolution estimations of cryo-EM maps for 80S ribosome PRE-H2 state with A/P A/A* tRNA. Fourier shell correlation (FSC) for cryo-EM maps of ribosomes, from two independent isolations (#1/#2), from wild-type (WT) or *ncs2Δelp6Δ* yeast. The global and local resolution indicated for final maps was estimated using a 0.143 FSC cut-off. (**E**) Global and local resolution estimations of cryo-EM maps for 80S ribosomes with P-site tRNA. Fourier shell correlation (FSC) for cryo-EM maps of ribosomes in the following states: PRE-translocation non-rotated P/P A/A (PRE-NR); PRE-translocation-hybrid P/E A/A (PRE-H1); post-translocation non-rotated E/E P/P (POST1-NR); post-translocation non-

rotated P/P (POST2-NR); and splitting complex (SC) with P/P tRNA, eRF1, and ABCE1. The global and local resolutions indicated for final maps was estimated using a 0.143 FSC cut-off.

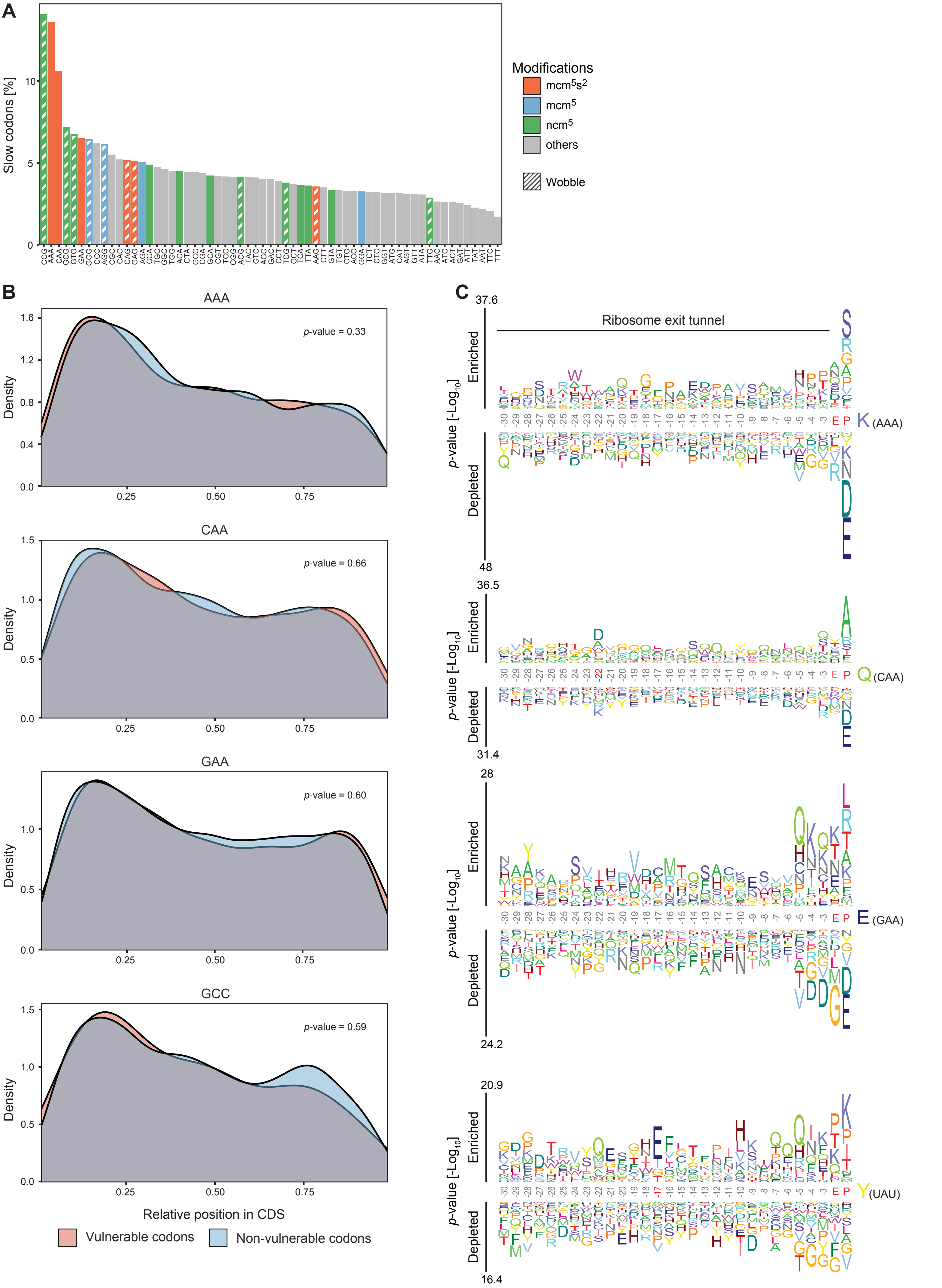

Supplementary Figure 3

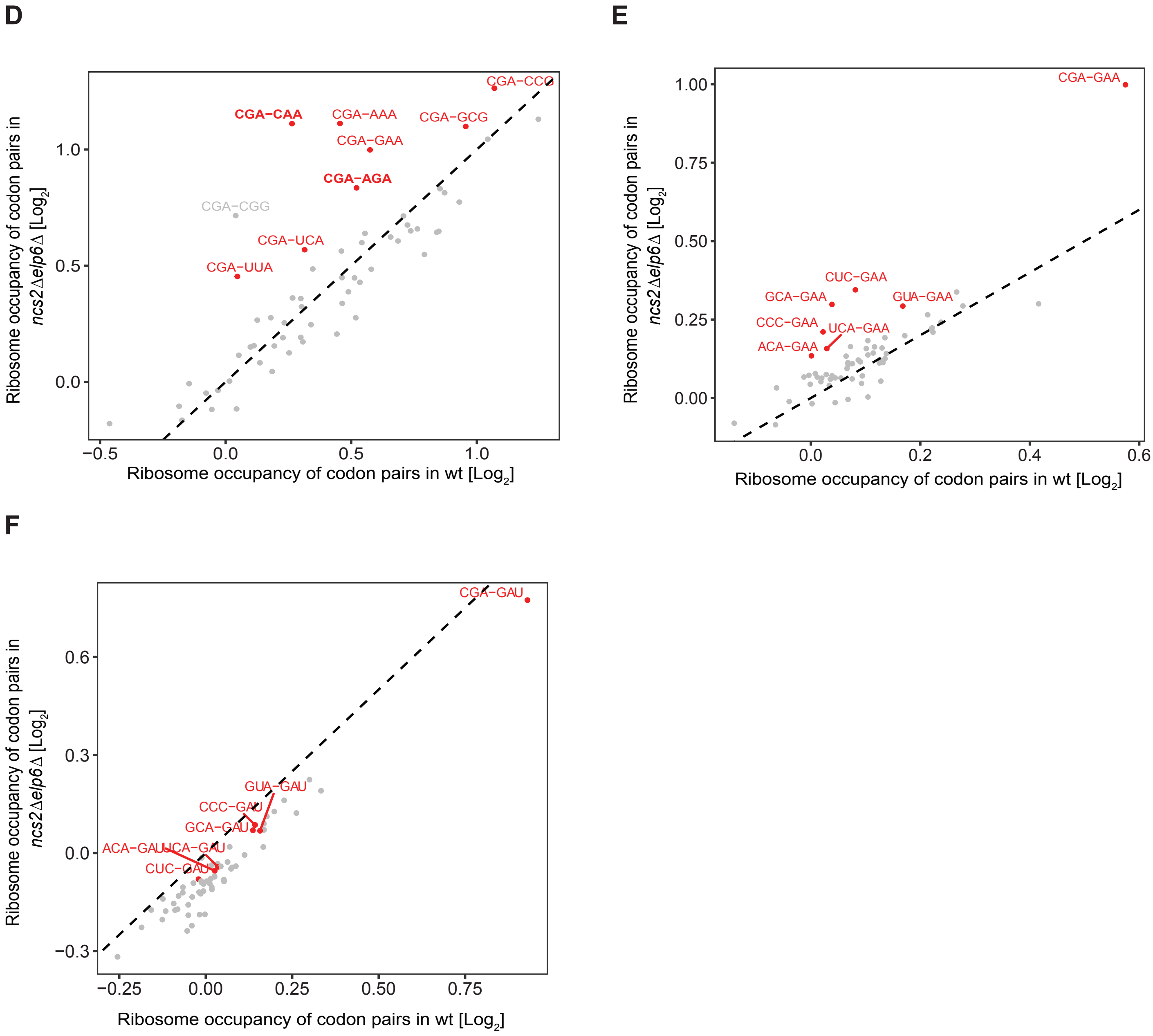

Supplementary Figure 3

Supplementary Figure 3. Strong pausing occurs at tRNA modification-dependent codon pairs. (**A**) The percentage of slow codons relative to all codons of the same instance across the transcriptome. Modification-dependent codons are highlighted by color: mcm^5s^2U (red); mcm^5U (blue); ncm^5U (green); wobble codons are indicated by diagonal lines. (**B**) Relative distribution across the CDS for vulnerable codons (red) and non-vulnerable codons (blue) for AAA, CAA, GAA and GCC (from top to bottom; difference analyzed by two-sided Kolmogorov-Smirnov test; *p*-values are indicated). For AAA: 1273 vulnerable codons, 2503 non-vulnerable codons; for CAA: 746 vulnerable codons, 1760 non-vulnerable codons; for GAA: 886 vulnerable codons, 7311 non-vulnerable codons; for GCC: 120 vulnerable codons, 1831 non-vulnerable codons. (**C**) Amino acid motifs upstream of AAA, CAA, GAA, and UAU codons (from top to bottom; ~30 codons in the ribosome exit tunnel) with high vulnerability scores (top 1000 codons are selected). Red letters or numbers indicate positions in the motif that are statistically significant (Bonferroni-corrected *p*-value < 0.01). (**D-E**) Relative ribosome occupancy of specific codon pairs between *ncs2*Δ*elp6*Δ and wild-type yeast. CGA-NNN (**D**) and NNN-GAA (**E**) codon pairs, and NNN-GAU (**F**) codon pairs that are not affected by U_{34} tRNA modifications are plotted. U_{34} modification-dependent codon pairs are highlighted in red in (**D**) and (**E**) but they are not slow in (**F**).

A

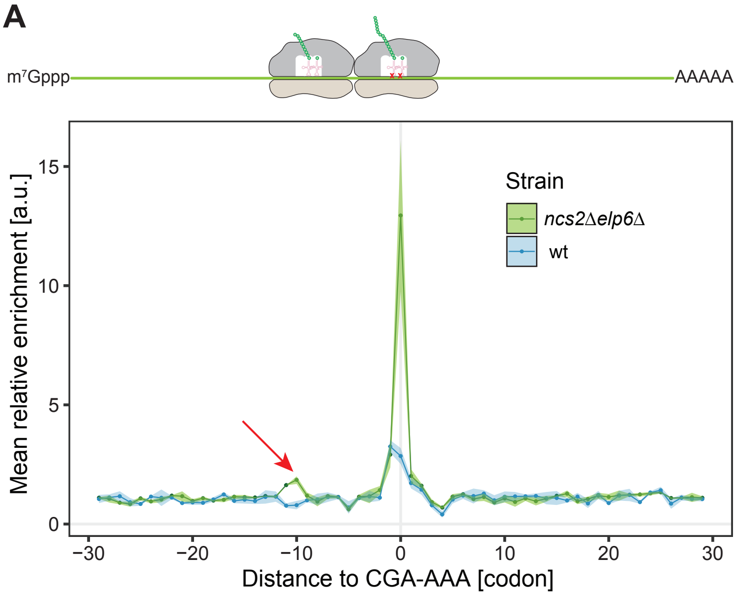

B

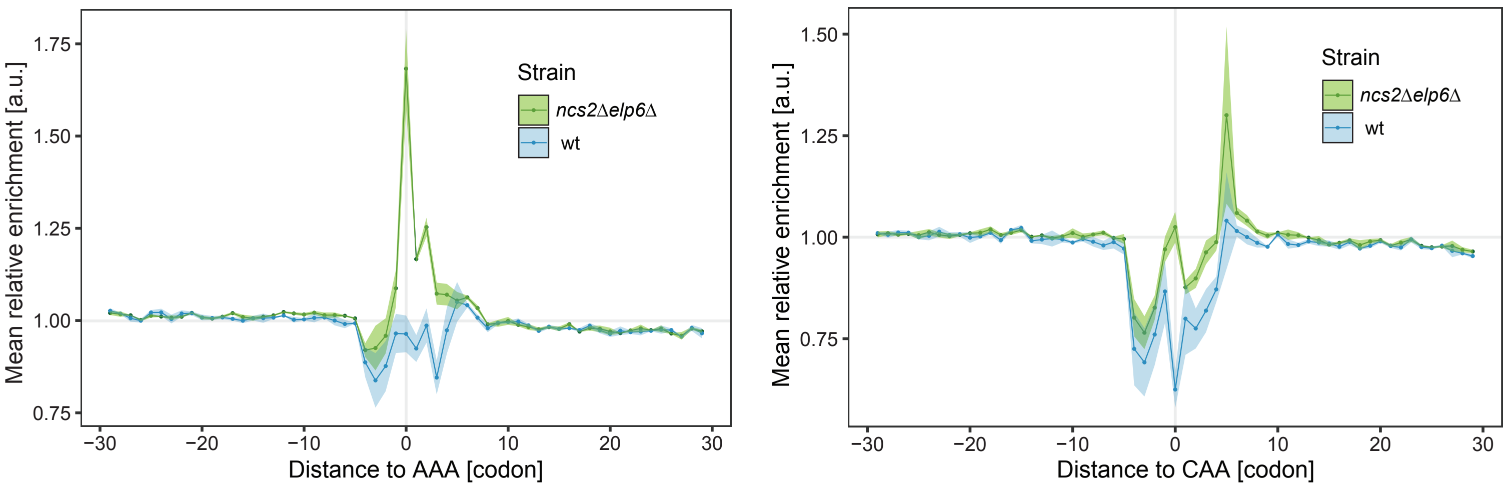

Supplementary Figure 4

Supplementary Figure 4. Ribosomes stall on slow codon pairs. (**A**) and (**B**) The distribution of ribosome occupancy (monosomes) around CGA-AAA (**A**), AAA (**B**, left), and CAA (**B**, right). Colliding ribosomes ~10 codons upstream of the codon pairs in the *ncs2*Δ*elp6*Δ mutant are highlighted by a red arrow. The shaded area indicates the degree of experimental variation within three replicates. The cartoon above (**A**) depicts collided ribosomes relative to the plot. Note that no queueing ribosomes are observed upstream of individual AAA and CAA codons. Three biological replicates were used for both genotypes.

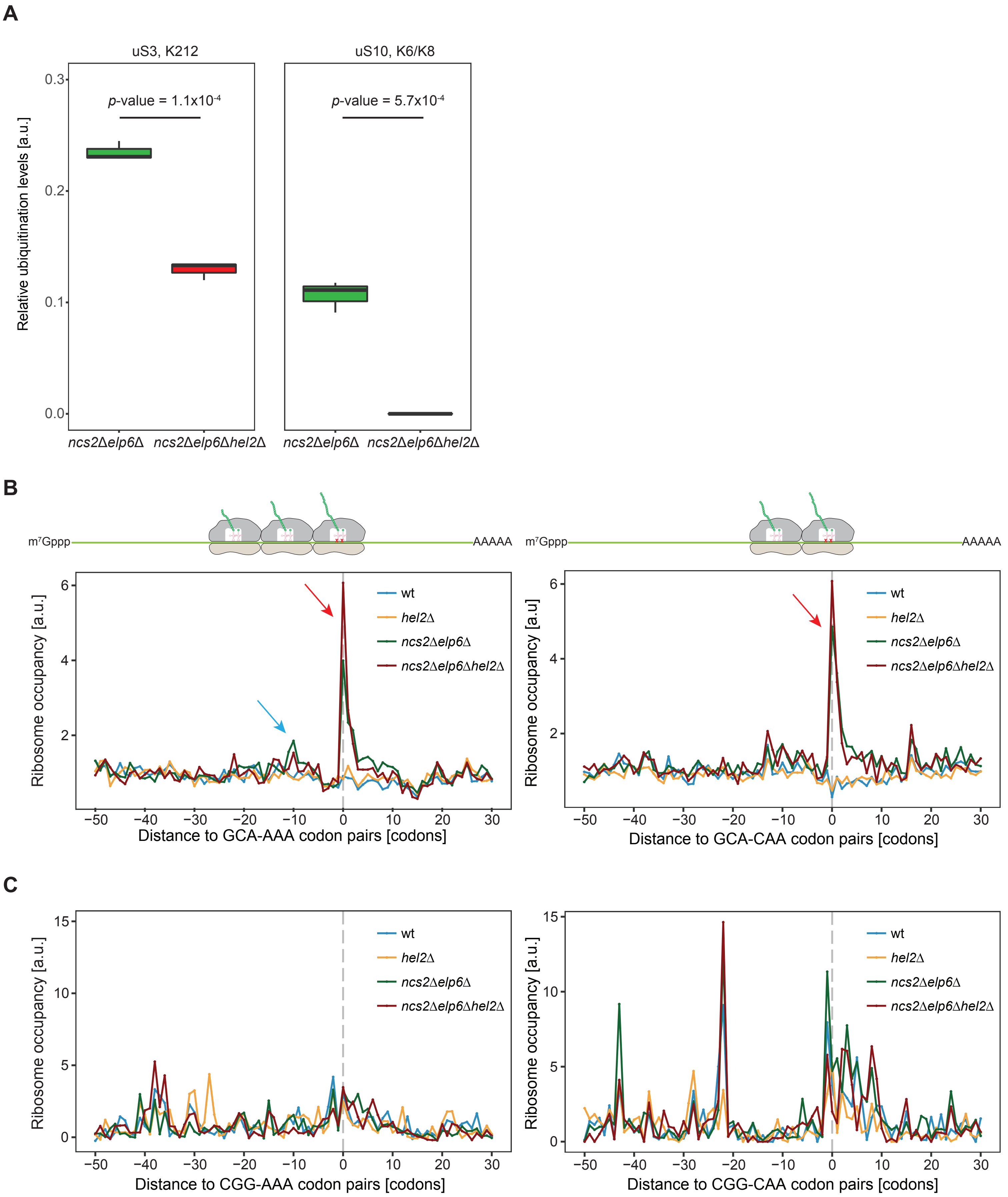

Supplementary Figure 5

D

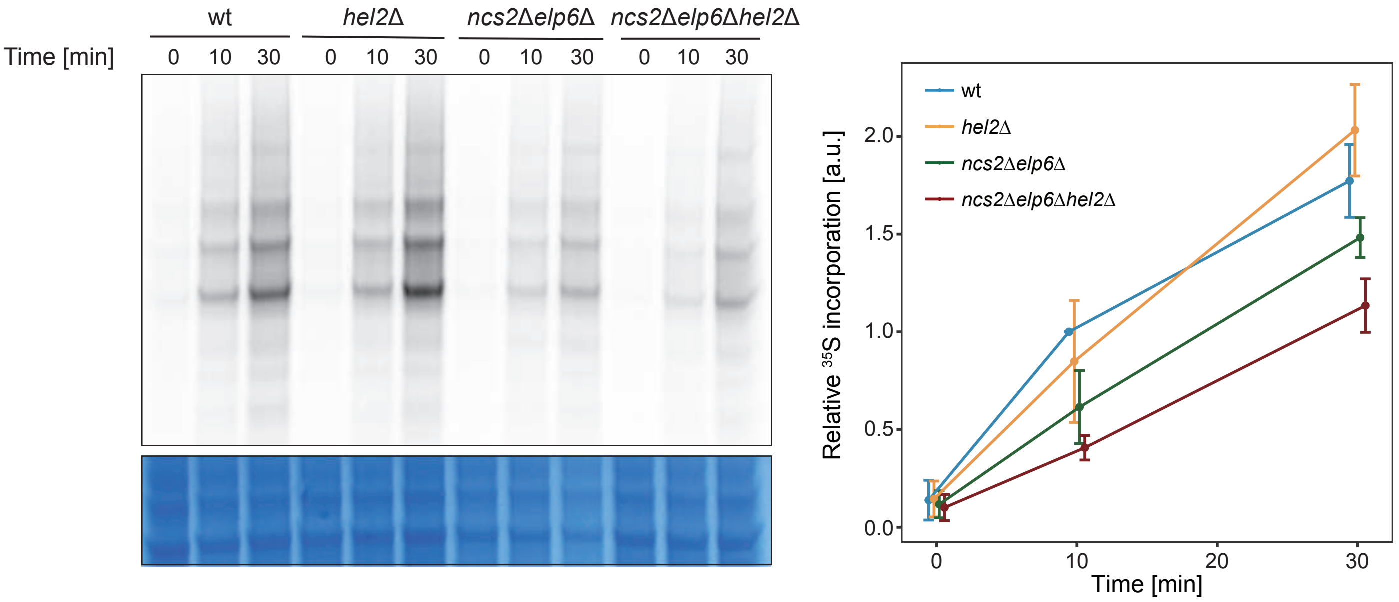

E

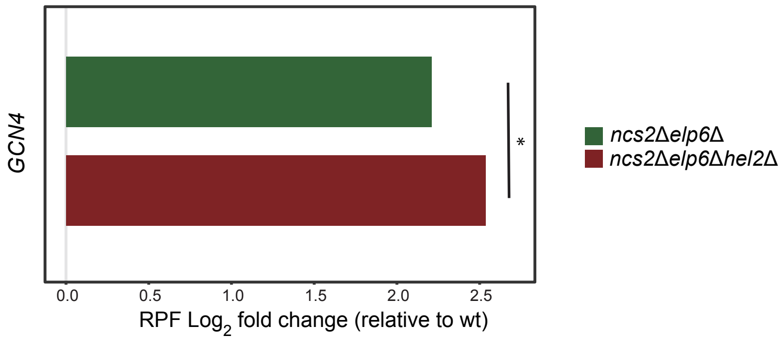

F

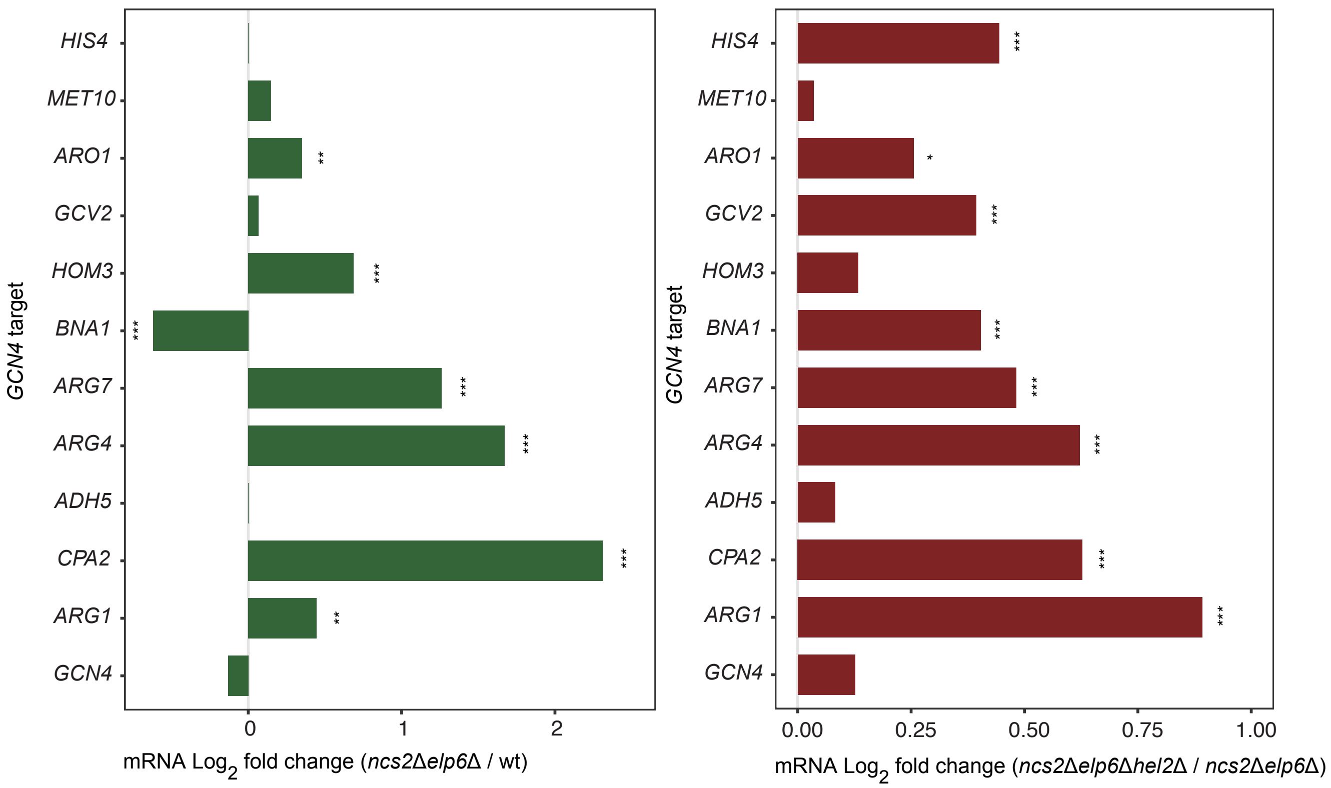

Supplementary Figure 5

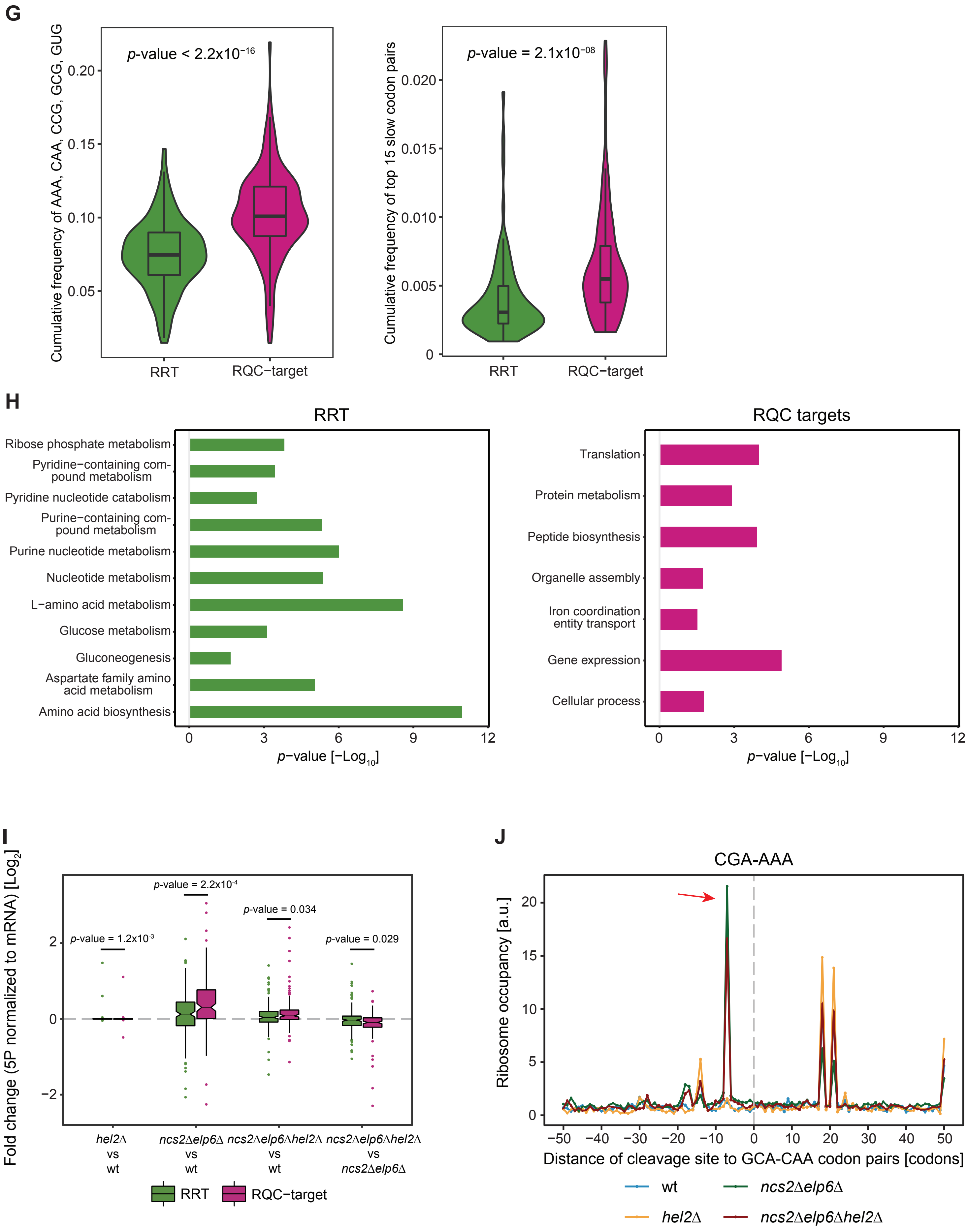

Supplementary Figure 5

Supplementary Figure 5. Ribosomes collide and are targeted by ribosome-associated quality control (RQC). (**A**) Quantification of ubiquitination levels of uS3 and uS10 by mass spectrometry in the *ncs2*Δ*elp6*Δ and *ncs2*Δ*elp6*Δ*hel2*Δ backgrounds (two-sided student's t-test). (**B**) Disome occupancy around GCA-AAA (left) and GCA-CAA (right) codon pairs in wild-type, *hel2*Δ, *ncs2*Δ*elp6*Δ and *ncs2*Δ*elp6*Δ*hel2*Δ yeast. The average disome occupancy from two biological replicates was plotted in (**B**) and (**C**). Peaks at position 0 represent the A site of the first stalling ribosome (red arrow). One queuing disome (blue arrow) was observed upstream of GCA-AAA, indicating a total of three ribosomes at the stalling site. The cartoon above depicts collided ribosomes relative to the plot. (**C**) Disome occupancy around the control codon pairs CGG-AAA and CGG-CAA in the same yeast strains. (**D**) Metabolic labeling of newly synthesized proteins after 0, 10, or 30 min incubation with ^{35}S L-methionine in wild-type, *hel2*Δ, *ncs2*Δ*elp6*Δ and *ncs2*Δ*elp6*Δ*hel2*Δ cells. Translation is strikingly impaired in *ncs2*Δ*elp6*Δ*hel2*Δ compared to *ncs2*Δ*elp6*Δ. A representative gel is shown on the left and quantification of three replicates is shown on the right. (**E**) Expression levels (RPF) of *GCN4* in *ncs2*Δ*elp6*Δ and *ncs2*Δ*elp6*Δ*hel2*Δ compared to wild type. (**F**) The transcription of Gcn4-targets was mostly upregulated in *ncs2*Δ*elp6*Δ yeast compared to wild type (left), and the upregulation is further increased in *ncs2*Δ*elp6*Δ*hel2*Δ (right). However, the transcription of *GCN4* itself does not change, indicating that the integrated stress response (ISR) is activated in the absence of ribosome-associated quality control (RQC) (two-sided student's t-test; **p*-value < 0.05, ***p*-value < 0.01; ****p*-value < 0.001). (**G**) The frequency of slow codons (left) or slow codon pairs (right) was compared between RQC-targets and RQC-refractory transcripts (RRTs) (one-sided Mann-Whitney U-test). (**H**) Gene ontology analysis of RRT (left) and RQC-targets (right). Significant terms in biological process are shown (Fisher's exact test). (**I**) Differential mRNA degradation analysis by 5PSeq in wild-type, *hel2*Δ, *ncs2*Δ*elp6*Δ and *ncs2*Δ*elp6*Δ*hel2*Δ cells. The analysis was performed by normalizing to mRNA level to exclude transcriptional effects. The degradation of RQC-target mRNAs was rescued in *ncs2*Δ*elp6*Δ*hel2*Δ compared to *ncs2*Δ*elp6*Δ yeast (two-sided Mann-Whitney U-test). (**J**) 5' ends of 5PSeq reads around CGA-AAA codon pairs (AAA codon at position 0) in wild-type, *hel2*Δ, *ncs2*Δ*elp6*Δ and *ncs2*Δ*elp6*Δ*hel2*Δ yeast; a strong peak indicates mRNA degradation upstream of CGA-AAA codon pairs (red arrow).

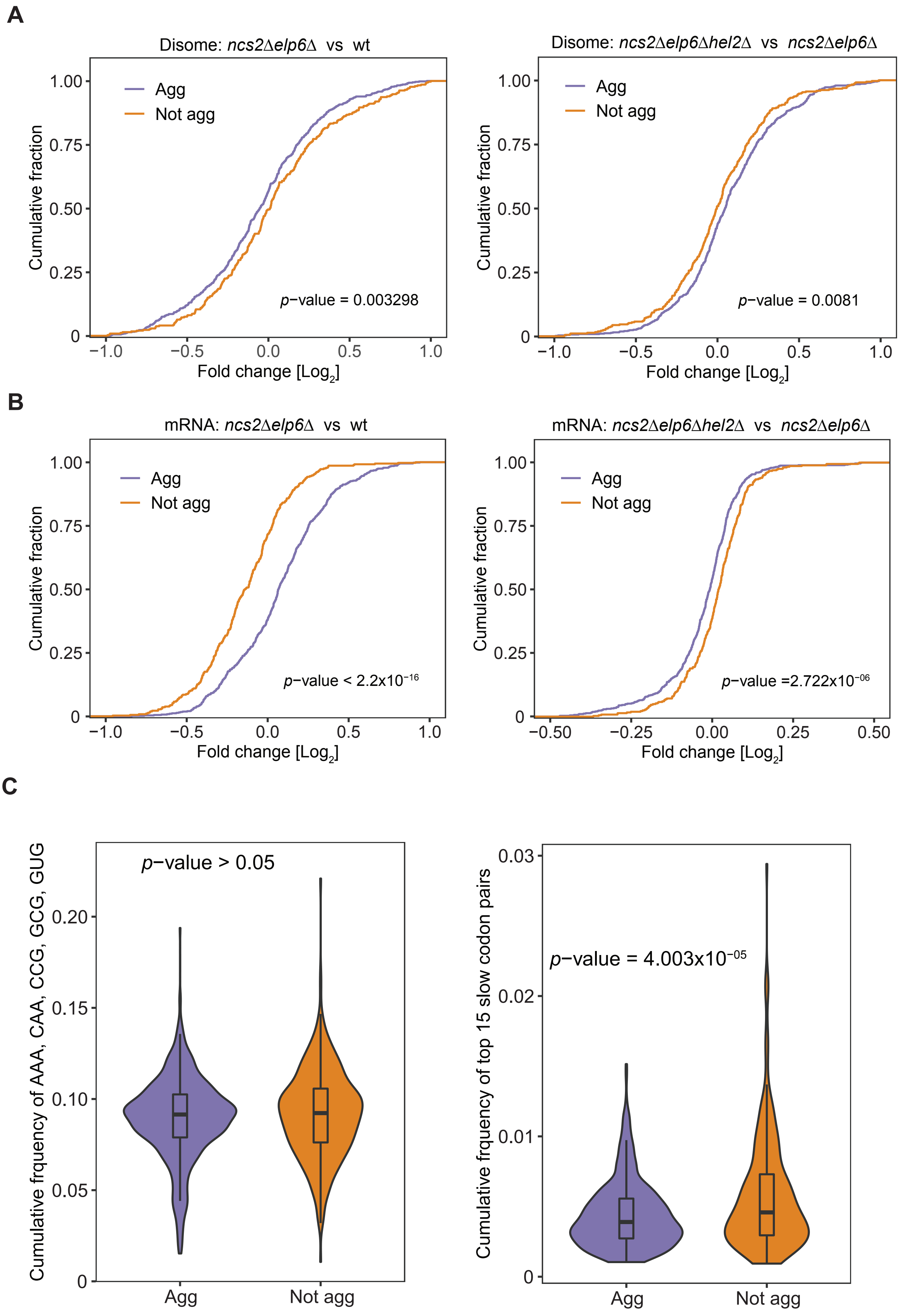

Supplementary Figure 6

D

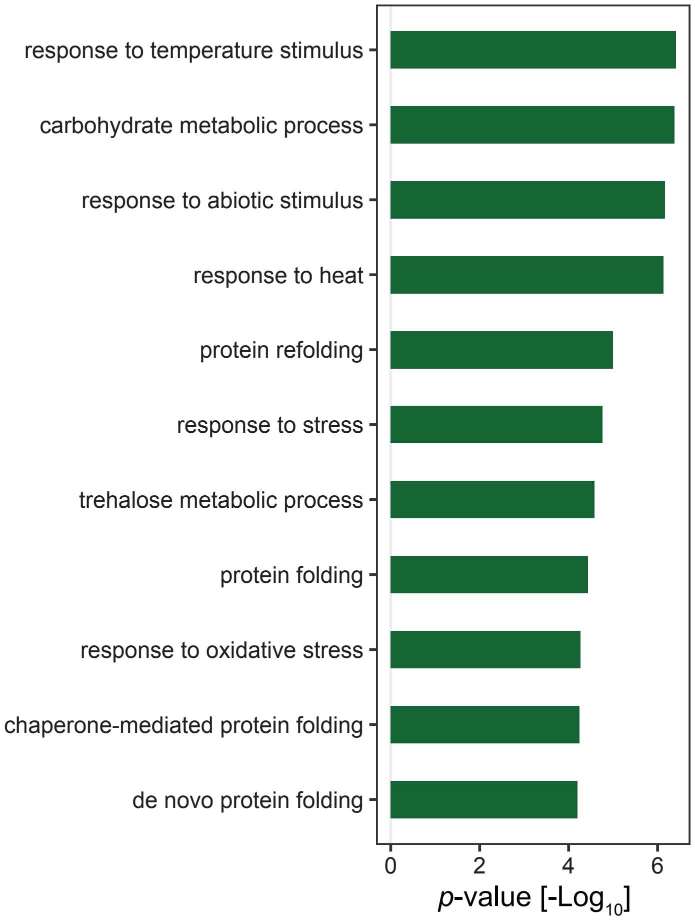

E

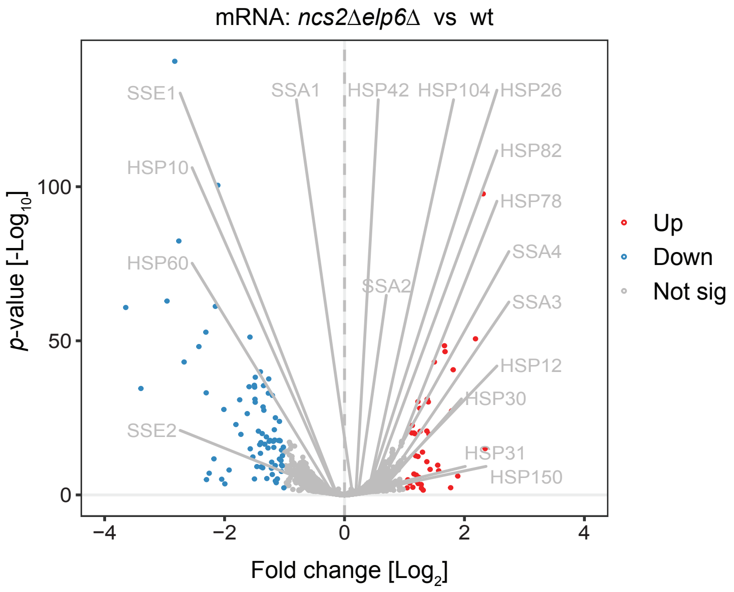

F

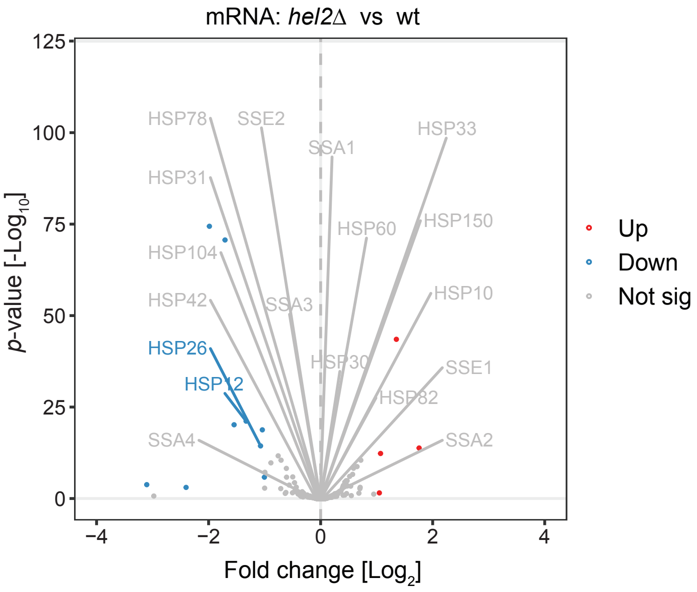

Supplementary Figure 6

Supplementary Figure 6. The RQC pathway alleviates protein homeostasis defects in coordination with chaperones. (**A**) The disome occupancy was compared between *ncs2*Δ*elp6*Δ and wild type (left) or between *ncs2*Δ*elp6*Δ*hel2*Δ and *ncs2*Δ*elp6*Δ (right) for aggregated (n=610) and non-aggregating (n=407) proteins using DESeq2 (3). Compared to aggregated proteins, disomes are more abundant on transcripts that encode non-aggregating proteins in the absence of U_{34} tRNA modifications (left) but are less abundant on these transcripts when *HEL2* is additionally deleted (right; one-sided Kolmogorov-Smirnov test). Monosome levels were used for normalization like for Figure 4b. (**B**) mRNA levels of non-aggregating proteins are lower in *ncs2*Δ*elp6*Δ yeast (left) and this effect is rescued in *ncs2*Δ*elp6*Δ*hel2*Δ cells (right) (one-sided Kolmogorov-Smirnov test). (**C**) The frequency of U_{34} modification-related slow codons (left) or codon pairs (right) were compared between aggregated proteins and non-aggregating proteins (one-sided Mann-Whitney U-test). (**D**) Gene ontology analysis of genes that are upregulated in *ncs2*Δ*elp6*Δ*hel2*Δ compared to *ncs2*Δ*elp6*Δ. Significant terms in biological process are shown (Fisher's exact test). (**E**) and (**F**) DESeq2 differential expression analysis of mRNA levels comparing *ncs2*Δ*elp6*Δ (**E**) and *hel2*Δ (F) to wild type, respectively. Chaperones are generally not altered in these two mutant strains. Up- or downregulated genes with p-adjusted value < 0.05 and Log_2 fold change > 1 are highlighted in red or blue.

Supplementary Material and Methods

Mass spectrometry:

50 OD_{600} units of yeast during exponential growth were harvested by vacuum filtering and snap-frozen with droplets of lysis buffer (4). The mixture of cells and buffer was lysed using a SamplePrep 6770 cryo-mill (Spex). The resulting powder was thawed in a water bath and cleared by 2 rounds of centrifugation. A_{260} absorbance of the lysate was measured, and 15 U of RNA were diluted 1:1 with 2 mM $CaCl_2$ and digested with 100 U Micrococcal Nuclease (Thermo Scientific) at 22 °C and 1'400 rpm for 15 min. Digested samples were loaded on a 10-50 % sucrose gradient and separated by ultracentrifugation at 35'000 rpm, 4 °C for 3 h using a SW41 rotor (Beckman Coulter). Sucrose gradients were fractionated using a Piston Gradient Fractionator (Biocomp). The disome peak was collected and proteins precipitated using TCA. Proteins captured on beads were subsequently processed for nano-liquid chromatography coupled to tandem mass spectrometric analysis on an Ultimate 3000 Orbitrap LUMOS (Thermo) using a Acquity CSH column (200 x 0.075 mm, 1.7µm; Waters) with a 90-min acetonitrile gradient (2-40%) at a flow rate of 220 nL/min.

The mass spectrometry data was first searched and quantified with FragPipe (5) version 18.0 against the *S. cerevisiae* swissprot (6) release January 2022, to which common contaminants were added. The precursor and fragment tolerance were set to 10 ppm and 0.4 Da, respectively. Search enzyme was set to trypsin, with a maximum number of allowed missed cleavages of 3. Carbamidomethylation on cysteine was set as a fixed modification; methionine oxidation and protein N-terminal acetylation were given as variable modifications. The minimum of matched fragments was set to 6. Validation was performed with the Peptide Prophet option, and a protein false discovery rate of 0.01. Proteins with less than 2 identified peptides were first removed from the list. A reduced database of those proteins positively identified by FragPipe was then used to search the samples with the following search engines: Comet (7), Xtandem (8), MSGF (9), Myrimatch (10) and MSFragger (11). Search parameters were same as above, with the addition of ubiquitinylation residue on Lysine as variable modification and a maximum of 4 modifications per peptide. Each search was followed by the application of the PeptideProphet (12) tool from the Transproteomics pipeline (TPP) (13), followed by the application of iprophet (14) from TPP to combine the search results, which were filtered at the false discovery rate of 0.01. The identification was only accepted if at least three of the search engines agreed on the identification. Protein inference was performed with ProteinProphet (15) from TPP. For those protein groups accepted by a false discovery rate filter of 0.01, a Normalized Spectral

Abundance Factor (NSAF) (16) was calculated based on the peptide to spectrum match count; shared peptides were accounted for according to (17).

References:

1. Hussmann JA, Patchett S, Johnson A *et al*. Understanding biases in ribosome profiling experiments reveals signatures of translation dynamics in yeast. PLoS Genet 2015;11:e1005732. https://doi.org/10.1371/journal.pgen.1005732
2. Johansson MJO, Esberg A, Huang B *et al*. Eukaryotic wobble uridine modifications promote a functionally redundant decoding system. Mol Cell Biol 2008;28:3301–12. https://doi.org/10.1128/MCB.01542-07
3. Love MI, Huber W, Anders S. Moderated estimation of fold change and dispersion for RNA-seq data with DESeq2. Genome Biol 2014;15:550. https://doi.org/10.1186/s13059-014-0550-8
4. Kim Y, Eggers C, Shvetsova E *et al*. Analysis of codon-specific translation by ribosome profiling. Methods Enzymol 2021;658:191–223.
5. Yu F, Haynes SE, Teo GC *et al*. Fast Quantitative Analysis of timsTOF PASEF Data with MSFragger and IonQuant. Mol Cell Proteomics 2020;19:1575–1585. https://doi.org/10.1074/mcp.TIR120.002048.
6. The UniProt Consortium. UniProt: a worldwide hub of protein knowledge. *Nucleic Acids Res 2019;*47:D506–D515. https://doi.org/10.1093/nar/gky1049.
7. Eng JK, Hoopmann MR, Jahan TA *et al*. A Deeper Look into Comet-Implementation and Features. *J Am Soc Mass Spectrom* 2015;26: 1865–1874. https://doi.org/10.1007/s13361-015-1179-x.
8. Craig R and Beavis RC. A method for reducing the time required to match protein sequences with tandem mass spectra. *Rapid Comm Mass Spectrometry* 2003;17: 2310-2316. https://doi.org/10.1002/rcm.1198.
9. Kim S and Pevzner PA. MS-GF+ makes progress towards a universal database search tool for proteomics. *Nat Commun* 2014;5:5277. https://doi.org/10.1038/ncomms6277.
10. Tabb DL, Fernando CG and Chambers MC. MyriMatch: Highly Accurate Tandem Mass Spectral Peptide Identification by Multivariate Hypergeometric Analysis. *J Proteome Res* 2007;6: 654-661. https://doi.org/10.1021/pr0604054.
11. Kong AT, Leprevost FV, Avtonomov DM *et al*. MSFragger: ultrafast and comprehensive peptide identification in mass spectrometry-based proteomics. *Nat Methods* 2017;14:513-520. https://doi.org/10.1038/nmeth.4256.
12. Choi H, Ghosh D and Nesvizhskii AI. Statistical Validation of Peptide Identifications in Large-Scale Proteomics Using the Target-Decoy Database Search Strategy and Flexible Mixture Modeling. *J Proteome Res* 2008;7:286-292. https://doi.org/10.1021/pr7006818.
13. Deutsch EW, Mendoza L, Shteynberg D *et al.* A guided tour of the Trans-Proteomic Pipeline. *Proteomics* 2010;10:1150-1159. https://doi.org/10.1002/pmic.200900375.

14. Shteynberg D, Deutsch EW, Lam H *et al.* iProphet: Multi-level Integrative Analysis of Shotgun Proteomic Data Improves Peptide and Protein Identification Rates and Error Estimates. *Mol Cell Proteomics* 2011;10:M111.007690. https://doi.org/10.1074/mcp.M111.007690.
15. Nesvizhskii AI, Keller A, Kolker E *et al.* A Statistical Model for Identifying Proteins by Tandem Mass Spectrometry. *Anal Chem* 2003;**75:**4646-4658. https://doi.org/10.1021/ac0341261.
16. Zybailov BL, Florens L and Washburn MP. Quantitative shotgun proteomics using a protease with broad specificity and normalized spectral abundance factors. *Mol BioSyst* 2007;3:354. https://doi.org/10.1039/b701483j.
17. Zhang Y, Wen Z, Washburn MP *et al.* Refinements to Label Free Proteome Quantitation: How to Deal with Peptides Shared by Multiple Proteins. *Anal Chem* 2010;82:2272-2281. https://doi.org/10.1021/ac9023999
